# TMTP1-modified Indocyanine Green-loaded Polymeric Micelles for Targeted Imaging of Cervical Cancer and Metastasis Sentinel Lymph Node* in vivo*

**DOI:** 10.7150/thno.35346

**Published:** 2019-09-25

**Authors:** Rui Wei, Guiying Jiang, MengQin Lv, Songwei Tan, Xueqian Wang, Ying Zhou, Teng Cheng, Xueqin Gao, Xi Chen, Wei Wang, Chenming Zou, Fei Li, Xiangyi Ma, Junbo Hu, Ding Ma, Danfeng Luo, Ling Xi

**Affiliations:** 1 Department of Obstetrics & Gynecology, Tongji Hospital, Tongji Medical College, Huazhong University of Science & Technology, Wuhan, People's Republic of China; 2 Cancer Biology Research Center (Key Laboratory of the Ministry of Education), Tongji Hospital, Tongji Medical College, Huazhong University of Science & Technology, Wuhan, People's Republic of China; 3 Tongji School of Pharmacy, Tongji Medical College, Huazhong University of Science & Technology, Wuhan, People's Republic of China; 4 Department of Thyroid and Breast Surgery, Tongji Hospital, Tongji Medical College, Huazhong University of Science & Technology, Wuhan, People's Republic of China

**Keywords:** TMTP1, metastasis, near-infrared imaging, sentinel lymph node, micelles, cervical cancer

## Abstract

Metastasis is one of the most threatening aspects of cervical cancer. We developed a method to intraoperatively map the primary tumor, metastasis and metastatic sentinel lymph nodes (SLNs), providing real-time intraoperative guidance in cervical cancer.

**Methods**: TMTP1, a tumor metastasis targeting peptide, was employed to modify the indocyanine green (ICG)-loaded poly (ethylene glycol)- poly (lactic-co-glycolic acid) (PEG-PLGA) micelles. The cervical cancer subcutaneous tumor model and lung metastasis model were established to determine the active targeting of ICG-loaded TMTP1-PEG-PLGA micelles (ITM) for the primary tumor and occult metastasis of cervical cancer. Human cervical cancer HeLa cells engineered by firefly luciferase were injected into the right hocks of BALB/c nude mice to develop the SLN metastasis model. The ITM and control ICG-loaded PEG-PLGA micelles (IM) were injected into the right hind footpads in the SLN metastasis model, and the migration and retention of micelles were recorded under near-infrared fluorescence. K14-HPV16 transgenic mice were also used to detect the image capability of ITM to target cancerous lesions.

**Results**: ITM could actively target imaging of the primary tumor and cervical cancer metastasis. ITM quickly diffused from the injection site to SLNs along lymphatic capillaries and remained in the SLNs for 12 h. Moreover, ITM specifically accumulated in the tumor metastatic SLNs (T-SLNs), which could be successfully distinguished from normal SLNs (N-SLNs).

**Conclusion**: ITM could achieve active targeting of the primary tumor, metastasis and T-SLNs, providing precise and real-time intraoperative guidance for cervical cancer.

## Introduction

Cervical cancer (CC) is one of the most common gynecologic tumors and the main cause of cancer death among young women worldwide 1. Approximately 46.4% of CC patients have a five-year survival rate of 91.5% after standard treatment. However, in the event of lymph node (LN) metastasis and distant metastasis, the five-year survival rate drops to 57.4% and 16.5%, respectively 2. Metastasis is the most important cause of CC death. Due to metastatic heterogeneity, the choices of effective treatment of metastatic CC are very limited. Therefore, accurate staging of CC in the early stages and timely and efficient diagnosis of metastasis can fundamentally improve the prognosis of CC and change the current dilemma of cancer treatment.

Magnetic Resonance Imaging (MRI) and Positron Emission Tomography-Computed Tomography (PET-CT) are used to detect relevant prognostic factors such as tumor size, parametrial infiltration, and lymph node (LN) status 3. However, no clinical data have confirmed that PET imaging has a reference value in the precise "T" staging of primary CC 4. The role of conventional MRI in the grading of LN metastasis may be unreliable because there is no consistent morphological feature to distinguish between benign and malignant LNs. In addition, these imaging modalities are mainly used for preoperative examinations. Translating these images into intraoperative images is very challenging and cannot always correspond to the findings during surgery 5. Therefore, diagnostic imaging agents are still urgently needed to accurately provide real-time images of CC metastasis and metastatic LNs during surgery.

Imaging-guided surgery with fluorescent probes has been intensively studied due to its simplicity and adaptability 6. Moreover, real-time intraoperative near-infrared (NIR) imaging is recognized as a promising technique for tumor imaging due to its characteristics of deep tissue penetration and low tissue auto-fluorescence 7. NIR fluorescence dyes, such as indocyanine green (ICG) approved by the United States Food and Drug Administration (FDA), have been used in clinical diagnostic applications including sentinel lymph nodes (SLNs) navigation during surgery 8. However, ICG imaging of LNs is nonspecific, and it is impossible to distinguish metastatic LNs from inflammatory LNs; thus, the false positive rate of imaging tumor metastases is high. In addition, several intrinsic limitations of ICG, including poor aqueous stability *in vitro* and low penetration depth, influence its imaging effect. Moreover, lack of reactive groups in ICG makes it difficult to further chemically synthesize with other agents such as targeted polypeptides 9.

The development of nanotechnology offers more possibilities for imaging and treatment of tumors. For example, Kanyi Pu ect have introduced semiconducting polymer nanoparticles for optical imaging in living mice^10^, ^11^. Advances in nanomedicine also provide more chances for the clinical applications of ICG and have showed promising results in preclinical models 12. In addition to passive targeting-induced by high permeability and retention effects of tumor tissue, nanoparticles can be modified with tumor targeting ligands (such as targeted peptides) on the surface to achieve active targeting. Although the advances of nanomedicine may provide a promising strategy for the imaging of tumors, traditional targeted delivery systems cannot effectively target tumor metastasis. Due to the existence of tumor heterogeneity, that is, the difference in gene expression profile, network regulation, mutation spectrum between tumor cells in different patient individuals or in different parts of the same patient, it brings great difficult to individualized imaging of tumor metastasis 13. Few studies achieved success in the active targeting of tumor metastasis 14. Most imaging agents show very low accumulation and have poor diagnostic efficacy for tumor metastases. Therefore, searching for a nanodelivery system that specifically targets tumor metastases is essential for imaging and further treatment.

The TMTP1 polypeptide is the five amino acid sequence NVVRQ obtained by our group using the bacterial flagellin library display technology. Our previous study verified that TMTP1 could target the primary highly metastatic tumor and the micrometastases. TMTP1 also specifically recognized atypical liver micrometastases that contained infrequent neoplastic cells 15. Binding peptides and fusion proteins prepared by combining TMTP1 with apoptotic peptides or diphtheria toxin could specifically inhibit tumor metastasis 16, 17. 99mTechnetium labeled with TMTP1 can target highly metastatic ovarian tumors 18. In addition, our study also revealed that XPNPEP2, a subtype of aminopeptidase P, is the potential receptor of TMTP1 15. Furthermore, we showed XPNPEP2 expression was obviously upregulated in CC tissue and that overexpression of XPNPEP2 was correlated with lymph node metastasis and worse overall survival 19. Therefore, TMTP1 polypeptide can be used to develop new diagnostic agents for the CC metastasis, especially micrometastasis.

For CC metastasis targeted imaging, in this study, we synergistically combined the tumor metastasis targeting peptide TMTP1 with ICG-loaded poly (ethylene glycol)- poly (lactic-co-glycolic acid) (PEG-PLGA) micelles. PEG-PLGA is a very promising biodegradable polymer, owing to its biocompatibility, biodegradation and sustained drug release profile 20. We observed the characteristics of micelles such as stability, size/charge changes and cellular uptake *in vitro*. Additionally, we provided the first demonstrations of *in vivo* tumor metastasis targeting and SLN mapping, particularly tumor metastatic SLNs *in vivo*.

## Results

### Preparation and characterization of ICG-loaded TMTP1-PEG-PLGA micelles (ITM)

**Figure 1:**
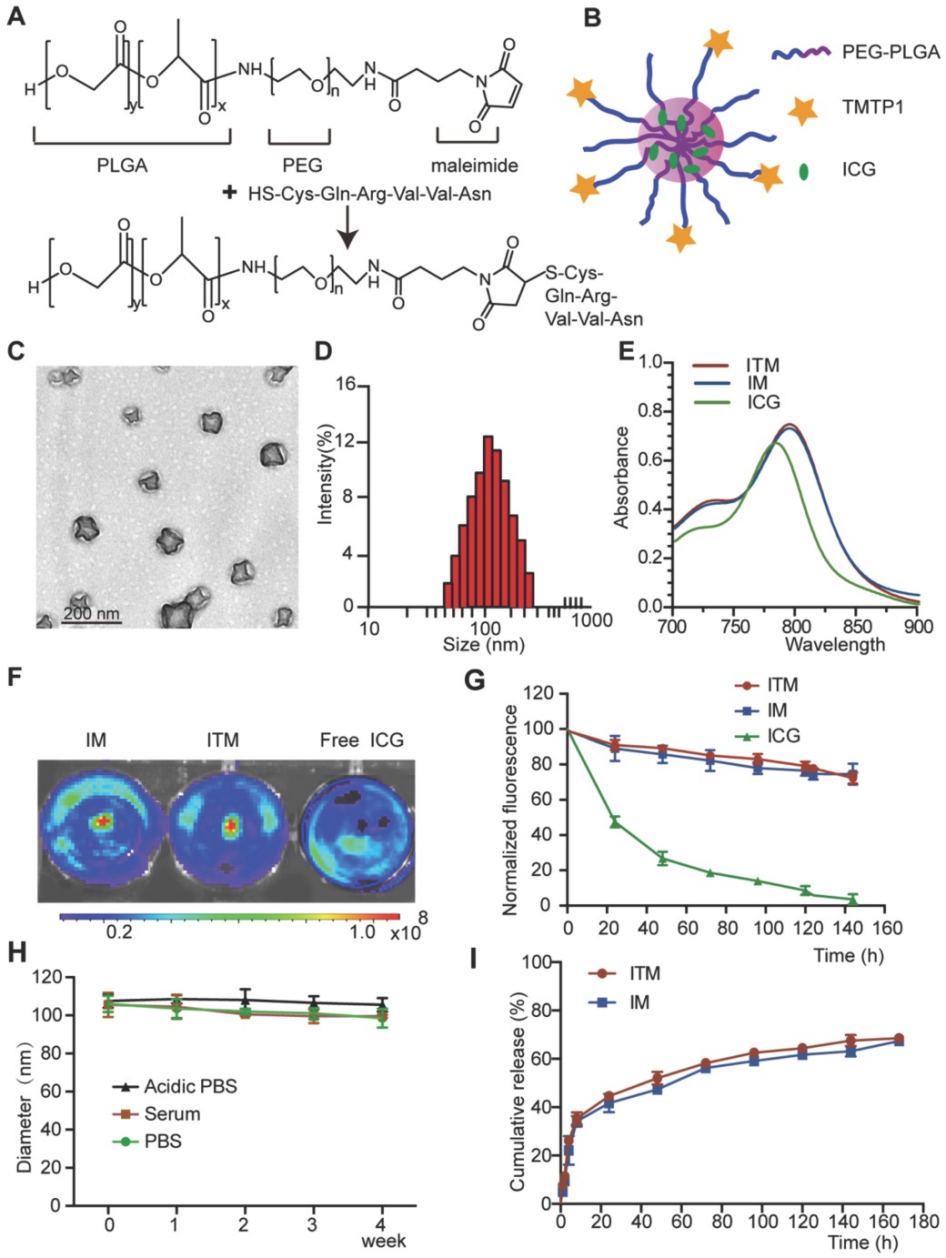
The preparation and characterization of TMTP1-modified ICG-loaded Polymeric Micelles. (A) ITM was synthesized by coupling the thiol group of TMTP1 with the maleimide group of PLGAPEG-MAL. (B) Scheme showing the structure of ITM. (C) TEM images of ITM. (D) The size distribution of ITM by DLS. (E) UV/Vis spectra of free ICG, IM and ITM in PBS. (F) NIR fluorescence images of ICG molecules in IM, ITM and free ICG at the same concentration (10 μg/mL). The fluorescence signals were acquired at 840 nm with the excitation of 745 nm. (G) Visible light exposure on the ICG degradation in the ITM、IM and the free ICG at the same concentration (10 μg/mL). The fluorescence intensity of each sample was measured using a spectrofluorometer with excitation and emission wavelengths of 745 nm and 840 nm, respectively. (H) Size stability test of ITM stored in serum, in phosphate-buffered saline (PBS, pH=7.4) or in Acidic PBS (pH=5.4) in the dark at 25 °C. (I) Drug release profiles of ITM or IM in PBS (pH=7.4) containing 10% FBS at 37 °C. (ICG: indocyanine green; ITM: ICG-loaded TMTP1-PEG-PLGA micelles; IM: ICG-loaded PEG-PLGA micelles.

The ITM was prepared by one-step nanoprecipitation and was synthesized via thiol-maleimide click chemistry. In this reaction, the thiol group (-SH) of TMTP1 was coupled to the maleimide group of PLGA-PEG- MAL (Figure 1A). The synthesized PLGA-PEG-MAL was confirmed by the ^1^H NMR spectrum, as shown in Figure S1A. The ^1^H NMR spectrum of PLGA-PEG-MAL showed peaks at approximately 8 ppm, which was attributed to MAL, a peak at approximately 3.6 ppm, which was attributed to the ethylene groups of PEG, and a peak at approximately 1.55 ppm, which was attributed to the methyl groups of PLGA. As measured by High Performance Liquid Chromatography (HPLC) (Figure S1B-C), a high conjugation efficiency (over 99%) of TMTP1 with PEG was attained in this study. A schematic representation of ITM was shown in Figure 1B. The ITM and ICG-loaded PEG-PLGA micelles (IM) were homogeneous in gelatinous form (Figure S2A). The transmission electron microscopy (TEM) image showed that ITM had a sub-100 nm spherical shape with perfect dispersity (Figure 1C). The average hydrodynamic diameter and surface charges of ITM and IM detected by dynamic light scattering (DLS) were shown in Table 1. It was shown that ITM and IM had similar average diameters at approximately 100 nm with a narrow distribution (Figure 1D and Figure S2B-C). The zeta potentials of ITM and IM were between -9.04 mV and -14.66 mV, which indicated that the particles could retain sufficient stability in the aqueous dispersion and maintain sufficient colloidal stability. The ICG encapsulation efficiency of ITM was 52 ± 8.14%. Compared with free ICG, the peak absorbance of ITM and IM was red shifted from 780 nm to 810 nm, and there was a slight increase in absorption at the 810 nm wavelength due to the hydrophobic environment in which the ICG molecules resided 21, 22 (Figure 1E). Additionally, the noncovalent binding between ICG molecules and nanocarrier reduced the fluorescence quenching caused by self-aggregation of free ICG 23. NIR fluorescence images of ICG molecules in IM and ITM at the same concentration (10 μg/mL) showed that the two ICG-loaded micelles exhibited similar fluorescence intensity and were slightly stronger than the free ICG (Figure 1F).

To examine whether the encapsulation of the ICG within the PEG-PLGA micelles could enhance the photostability of the ICG fluorescence against external light, ITM and IM were exposed to visible light for a predetermined period, and the fluorescence intensity was analyzed. The ITM and IM exposed to the visible light maintained their fluorescence intensity over the 6-day period. However, after 1 day of exposure to visible light, the fluorescence intensity of the free ICG solution was reduced to less than 50% of its initial value and was less than 1/2 of ITM and IM (Figure 1G). The ITM and IM showed remarkable stability without significant size changes in PBS, acidic PBS (pH ∼ 5.0, mimicking the acidic microenvironment of the lysosome), and fetal bovine serum buffer for 4 weeks (Figure 1H and Figure S2D), which ensured their feasibility for use in* in vivo* applications. The* in vitro* release characteristics of ICG from ITM and IM were shown in Figure 1I. During the first 12 h, a rapid release occurred with a cumulative release of 30%, which was mainly attributed to the initial burst of free ICG in micelles. Subsequently, from 12 to 160 h, a relatively slow release occurred with a cumulative release up to 60%. The results indicated that ITM micelles achieved sustained release of ICG, which was a critical factor for its application in optical imaging. The *in vitro* cytotoxicity of ITM and IM was measured using the cell counting kit-8 (CCK-8) assay with cervical cells. The results shown in Figure S2E-F indicated that the cell viability of cervical cells was not altered and maintained at approximately 100% even at higher concentrations of ITM and IM, which indicated that ITM and IM were safe and nontoxic.

### *In vitro* cellular uptake

**Figure 2:**
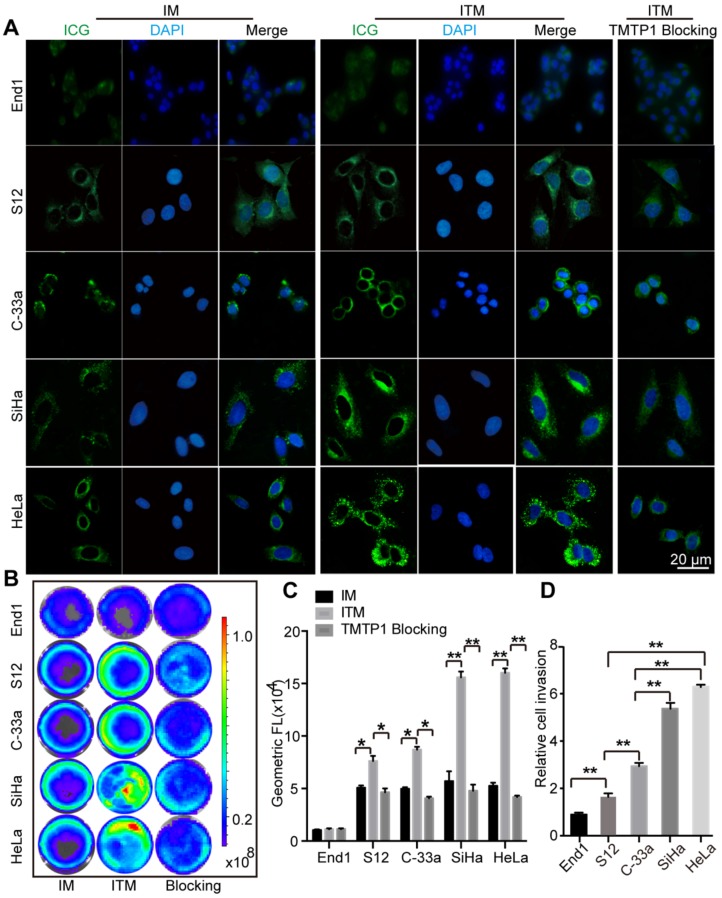
The *in vitro* cellular uptake. (A) Confocal fluorescence images of cervical cells treated with IM and ITM (ICG concentration = 10 μg/mL) for 3 h. For the *in vitro* competition assay, cells were pre-incubated with TMTP1 peptide for 30 min and then incubated with ITM for 3 h. Blue represents the fluorescence of DAPI and green represents the molecules fluorescence of ICG. The fluorescence images were obtained at excitation wavelengths of 405 and 633 nm for DAPI and ICG, respectively (scale bar, 20 μm). (B) NIR fluorescence images of ICG molecules from cervical cells incubated with the IM or ITM or preincubated with TMTP1 peptide for 30 min and then incubated with ITM (ICG concentration = 10 μg/mL). The NIR fluorescence signals were acquired at 840 nm with the excitation of 745 nm and were measured in radiance counts per cm^2^ per second per steradian (p/s/cm^2^/sr). (C) Flow cytometric analysis of mean fluorescence intensity of cervical cells incubated with IM or ITM or pre-incubated with TMTP1 peptide for 30 min and then incubated with ITM (ICG concentration = 10 μg/mL). (D) Statistical analyses of cellular migration of cervical cells. Data are expressed as mean ± s.e.m. (*P < 0.05, **P<0.01).

Our previous studies have demonstrated that HeLa and SiHa cells showed high expression of XPNPEP2, while C-33a and S12 cells expressed a relatively low level of XPNPEP2 by the immunoblotting assay compared with the normal cervical cell line End1 19. Cervical cells were treated with IM and ITM to detect the targeting of ITM (Figure 2A). The uptake of ITM by End1 was not significantly different from that of the control IM. Additionally, the uptake of ITM in S12 and C-33a was slightly stronger than that of IM, and for HeLa and SiHa, the fluorescence intensity of the cells treated with ITM was significantly stronger than that in the IM group. More particulate fluorescent substances were clearly observed in the cytoplasm of HeLa and SiHa incubated with ITM. To address the specificity of ITM for CC cells, we performed a competition assay in cervical cells. Cells were pretreated with excess TMTP1 peptide for 30 min and then incubated with 10 μg/mL ITM. The internalization of ITM into TMTP1-treated HeLa and SiHa cells was greatly inhibited compared with untreated cells. Quantitative analysis of the ICG fluorescence intensity in cervical cells was performed using ImageJ software (Figure S3A). The results suggested that ITM could bind specifically to CC cells through the ligand effects of TMTP1. NIR fluorescence images of cervical cells incubated with ITM or IM or preincubated with TMTP1 peptide for 30 min and then incubated with ITM (ICG concentration =10 µg/mL) at 37 °C for 3 h showed the same results (Figure 2B). Quantitatively, the fluorescence intensity of ITM was similar to that of IM in End1 cells, while the ICG fluorescence intensity for ITM compared with IM was enhanced approximately 1.31-fold in S12, 1.61-fold in C-33a, 2.32-fold in SiHa and 2.51-fold in HeLa cells (Figure 2C).

The fluorescence intensity of ITM was enhanced approximately 1.75-fold in S12, 1.91-fold in C-33a, 2.94-fold in SiHa and 3.13-fold in HeLa cells compared with the TMTP1 blocking group. The higher cellular uptake efficiency of ITM than IM in CC cells was probably due to the expression of XPNPEP2 receptors on CC cells. Interestingly, a stronger fluorescence signal was detected in HeLa and SiHa cells compared with C-33a and S12 cells when all of them were incubated with ITM. The difference in cellular uptake efficiency among these cells could rationally be interpreted as evidence for greater XPNPEP2 receptor expression on SiHa and HeLa than C-33a and S12 cells. In fact, we have demonstrated that TMTP1 binds to highly metastatic tumors, such as breast cancer MDA-MB-435S, but not to nonmetastatic cell lines (such as MCF-7) 15. Similarly, when compared to IM, ITM could specifically bind to MDA-MB-435S but not to MCF-7 (Figure S3B-C). Transwell assays showed that the invasive ability of SiHa and HeLa cells was higher than S12 and C-33a (Figure 2D). In comparision with C-33a and S12 cells, the stronger fluorescence signal in SiHa and Hela when all of them were incubated with ITM was consistent with the finding that TMTP1 targeted highly metastatic tumor cells. These results confirmed that the presence of TMTP1 on the surface of the carrier facilitated micelles uptake in CC cells, especially the highly metastatic tumor cells.

### *In vivo* distribution of ITM

**Figure 3:**
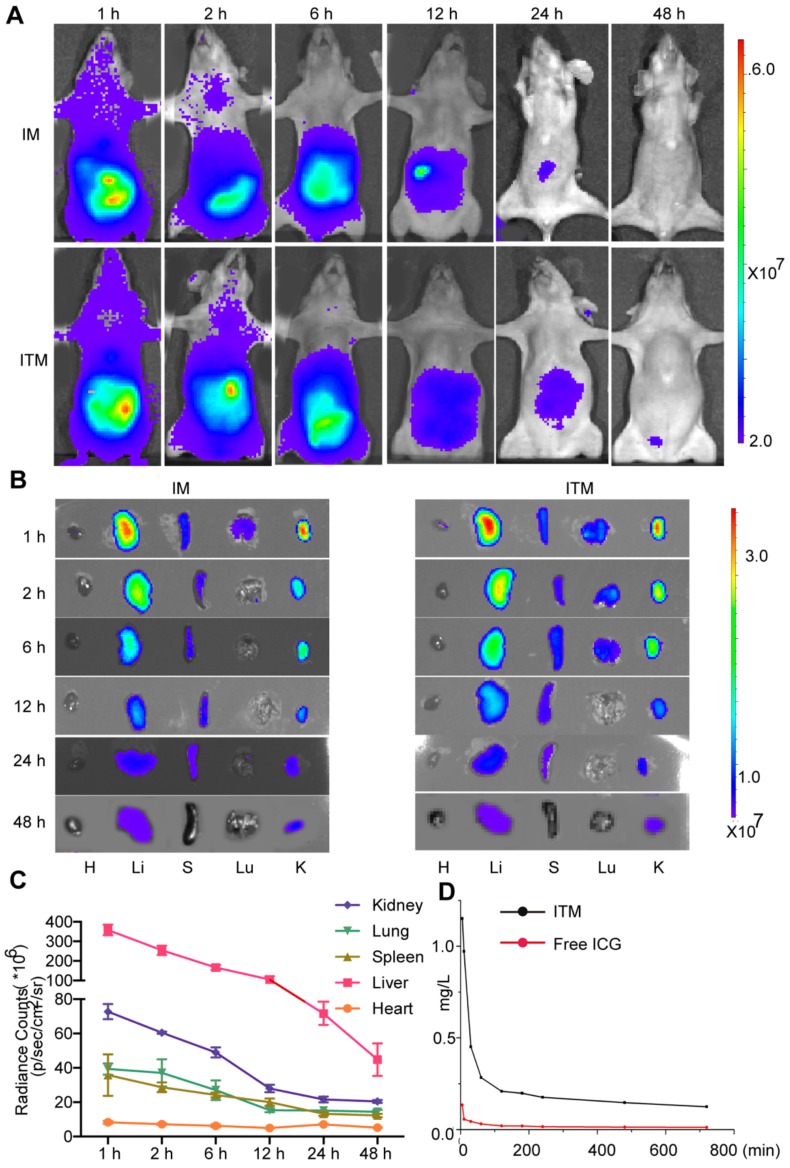
Dynamics and bio-distribution of ITM. (A) NIR fluorescence images of normal BALB/cnude mice after injection of ITM or IM at a dose of 1.0 mg /kg at different time points (1 h, 2 h, 6 h, 12 h, 24 h and 48 h). The fluorescence signals were acquired at 840 nm with the excitation of 745 nm and were measured in radiance counts per cm^2^ per second per steradian (p/s/cm^2^/sr). (B) NIR fluorescence images of the isolated major organs at the different time points. (C) Quantitative analysis of the fluorescence signal of the major organs of the mice received the injection of ITM at different time points. (D) ITM and free ICG concentration in the plasma (mg/L) of the BALB/c-null mice after injection of 10 μg ITM or free ICG.

To evaluate the metabolism of ITM* in vivo*, BALB/c normal mice were injected intravenously with ITM or IM and monitored for 48 h. The results showed that ITM and IM were dispersed throughout the body at 1 h post-injection, followed by accumulation mainly in the liver (Figure 3A). The overall fluorescence signal decreased with time, and the metabolism of ITM mainly occurred in the liver followed by the kidney, spleen and lung (Figure 3B-C). The liver, lung and spleen make up the reticulo-endothelial system (RES) in the body, which is responsible for removing foreign particles from the blood circulation, in accordance with the above observations 24. However, Studies have shown that nanoparticles will mainly undergo renal clearance when their size is smaller than 5.5 nm 10. The aggregation of ICG in kidney in our research was probably due to the release of free ICG from ITM in other tissues into the blood, resulting in the highest accumulation in the liver and kidney 25.

The concentration of ITM in plasma at 5 min was 1.151 µg/mL. The ICG concentration in the blood when delivered via PEG-PLGA micelles was significantly higher (8 times) than that administered as free ICG solution (Figure 3D). The half-life of ITM was 20 min, and the ITM concentration in the blood remained significantly high for 6 h. Table 2 summarizes the AUC _0-12 h_ in plasma. AUC represents the tissue exposure to the drug. The relatively long circulation of ITM in the blood might be attributed to the controlled release of ICG from ITM deposited in various organs within the body and modification of PEG on the micelle surface. These results suggested that ITM had a long circulation duration and good polymer degradation.

### Targeted delivery to tumor and tumor metastasis foci of cervical cancer *in vivo*

**Figure 4:**
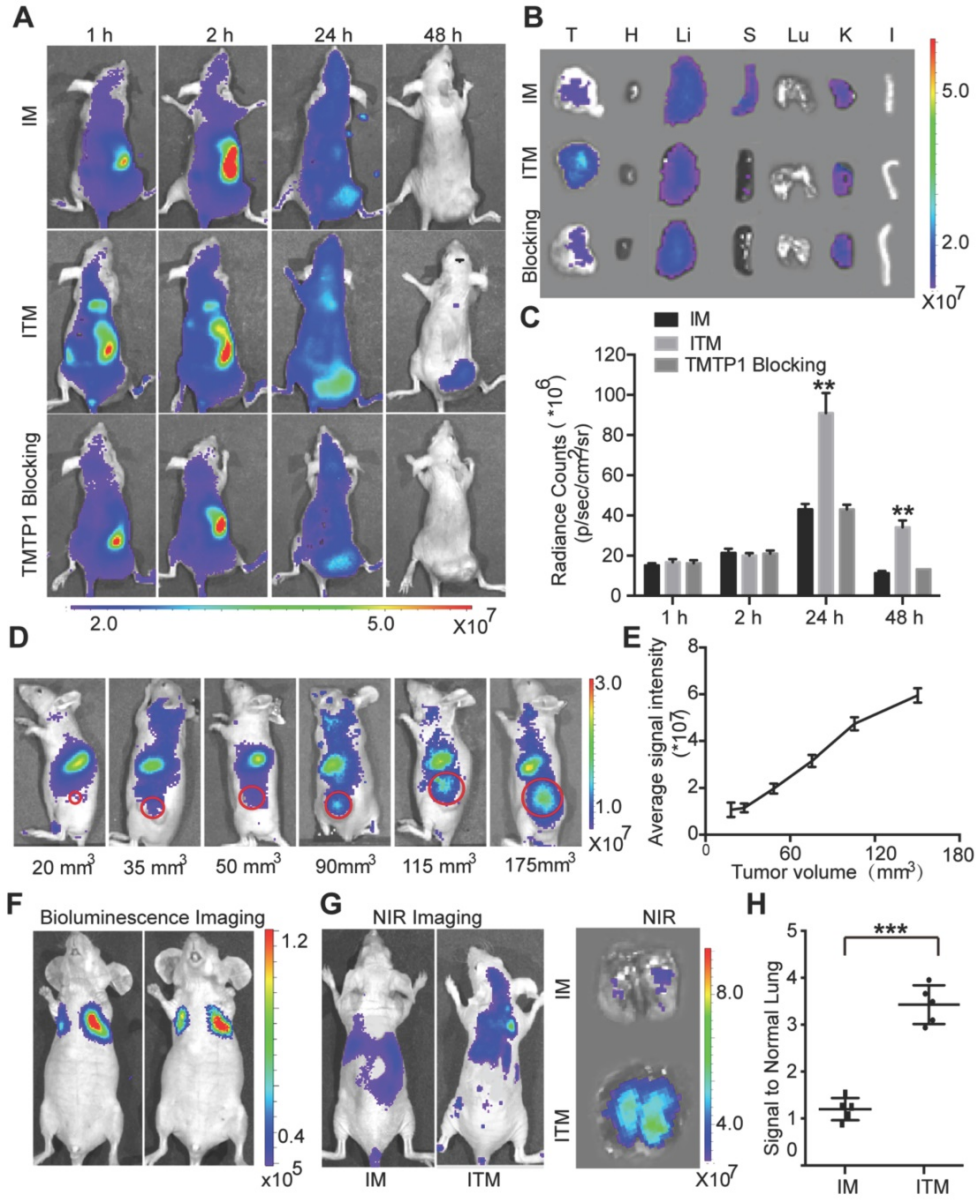
Targeted delivery to tumors and tumor metastases of cervical cancer *in vivo*. (A) Mice with subcutaneous xenografts derived from HeLa cells were injected intravenously with ITM or IM at an ICG dose of 1.0 mg/kg. Intravenous injection of 100 μl 100 μM TMTP1 before ITM was used as a competitive blocker. The fluorescence signals were acquired at 840 nm with excitation at 745 nm and were measured in radiance per cm^2^ per second per steradian (p/s/cm^2^/sr). (B) NIR fluorescence images of the isolated major organs and tumors at 48 h (T, tumor; H, heart; Li, liver; S, spleen; Lu, lung; K, kidney; I, intestine). (C) Average ICG fluorescence intensities of the tumors at the different time points (n = 5 per group). (D) ITM fluorescence imaging of mice with subcutaneous xenografts with different volumes. The fluorescence signals were acquired at 840 nm with excitation at 745 nm. (E) Average ICG fluorescence intensities of tumors with different volumes. (F) Bioluminescence imaging of lung metastatic foci in a lung metastasis model. Luciferase activity is measured in photons per cm^2^ per second per steradian (p/s/cm^2^/sr). (G) *In vivo and ex vivo* fluorescence imaging of lung metastatic foci at 24 h post-IM or ITM injection at an ICG dose of 1.0 mg/kg. The fluorescence signals were acquired at 840 nm with excitation at 745 nm. (H) Average ICG fluorescence intensities of lung metastatic foci at 24 h post-IM or ITM injection. (**P<0.01, ***P<0.001).

To demonstrate that ITM can target the primary tumor and metastasis of CC *in vivo*, HeLa cells were used to establish CC subcutaneous tumors and a pulmonary metastasis animal model. The NIR imaging results of the CC subcutaneous tumor model were presented in Figure 4A. When the mice were imaged in the initial hours after injection of ITM and IM, it was difficult to acquire clear tumor images because of the strong background signal from ICG in the liver and blood. After 24 h, the liver cleared part of the ICG, and the tumor was specifically visualized in the ITM group. The fluorescence signals were significantly stronger in the tumors of the ITM group compared with the mice that received IM at 24 h. ITM particularly exhibited a relatively long-term retention of detectable NIR signals in tumors during the imaging period of 48 h, while the NIR signal of IM in tumors clearly decreased with time after injection at 48 h. To further illustrate the distribution of ITM and IM in organs and tumor, the main organs, as well as tumor tissues, were excised for NIR ex-vitro imaging after injection at 2 h, 6 h, 12 h and 24 h (Figure S4). ITM resulted in continuous increases in the ICG concentration in tumor tissue from the beginning until the end of the study. After injection of ITM and IM at 24 h, the ICG concentration in tumor tissue of the ITM group was 1.21μg/g, more than 2 times than the IM group (0.49 μg/g). The tumor/liver ratio of ITM exceeded 1.0 between 12 and 24 h. In contrast, the ICG concentration in the ITM group was mildly increased within 12 hours, probably due to the EPR effect, and then it did not increase at 24 h, leaving few micelles in the tumor tissue. To evaluate the utility of active targeting, intravenous injection of TMTP1 before injection with ITM was used as a competitive blocker. TMTP1 exhibited an obvious blocking effect (Figure 4A). As shown in Figure 4B, higher fluorescence signals were observed in tumors from mice injected with ITM micelles compared with the IM group and TMTP1 blocking group. Quantitatively, ITM resulted in 3.12-fold higher accumulation in tumors compared with IM (Figure 4C). To further test the accumulation of micelles and gain insight into the mechanisms of accumulation, excised tumor tissues of mice after injection of ITM or IM were sectioned into 6-μm slices and then stained for XPNPEP2. ITM accumulated at higher levels than IM in the tumor tissue, which was consistent with the *in vivo* NIR imaging data. Immunofluorescence experiments showed that ITM mostly co-localized with XPNPEP2 and further demonstrated the plausibility of targeting tumor cells by TMTP1 (Figure S5). These results indicated that ITM could accumulate in tumors by active targeting and achieve good tumor localization for tumor imaging.

To demonstrate the sensitivity of ITM as the tumors grew to different sizes over time,the mice were inoculated varying cell numbers to form tumors with different sizes. ITM was administered through the tail vein (Figure 4D). Tumor imaging of ITM was positively correlated with tumor volume (Figure 4E). Nodules of 90 mm^3^ could be clearly identified by ITM. Despite the tendency of micelles to accumulate in the nodules of 35 mm^3^, the signal from the cells was not sufficiently strong to be separated from the background. For the *ex vivo* NIR imaging, the tumor tissues were excised after being imaged (Figure S6A).

HeLa-Luc cells with luciferase expression were injected intravenously to establish a lung metastasis model. After 3 weeks, luciferase bioluminescence was used to detecte the formation of lung metastatic foci (Figure 4F). At 24 h post-intravenous administration of ITM or IM into lung metastasis foci-bearing mice, NIR images of dissected lungs showed much stronger signals of ITM in lung metastases than that in the IM group (Figure 4G-H), confirming the active targeting of tumor metastases by ITM. To ascertain the time required for ITM targeting fluorescence to demonstrate lung metastasis formation after intravenous inoculation of HeLa-Luc cells, the mice were injected with ITM after inoculation of HeLa-Luc cells at 0, 1, 2 and 3 weeks. ITM could prominently accumulate in the lung metastasis foci after 2 weeks (Figure S6). Overall, ITM could actively accumulate in the primary tumor and metastasis of cervical cancer, which offered an opportunity for cancer metastasis-specific imaging and therapy.

Whether the incorporated drugs can be released from micelles is critical for carriers to successfully deliver them to targets. To clarify whether ICG could be released from ITM *in vivo*, we incorporated ICG into rhodamine B-labeled PLGA-PEG micelles and investigated their interactions after intravenous injection into HeLa tumor-bearing BALB/c nude mice at 6 h, 12 h and 24 h. The different localization patterns of rhodamine B-labeled PLGA-PEG and ICG in the tumors were shown in Figure S7. Real-time observations over a 24-h period showed increasing micelle accumulation in the tumor, and the red and green fluorescence were clearly separated at 24 h, indicating that PLGA-PEG and ICG were disassembled and ICG could be released from the PEG-PLGA micelles *in vivo*.

### *In vivo* NIR fluorescence LN imaging of normal mice

**Figure 5:**
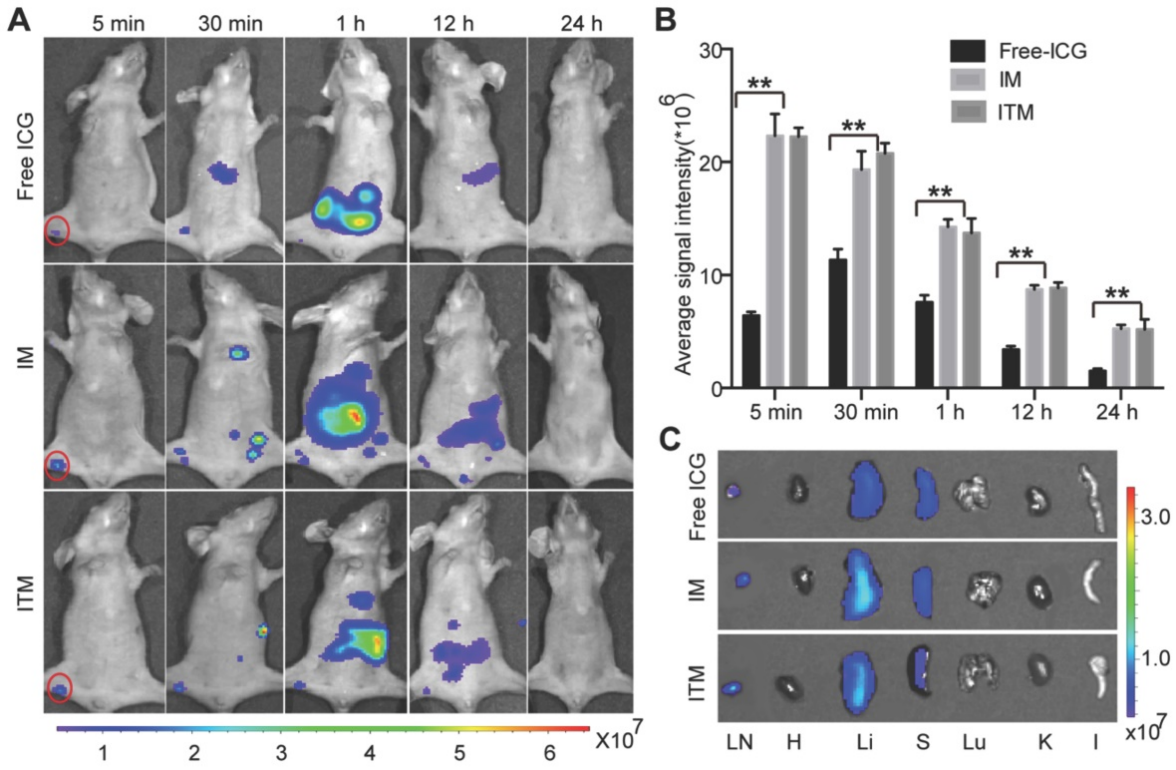
*In vivo* NIR fluorescence lymph node (LN) imaging of normal mice. (A) Optical imaging of LNs after injection of IM, ITM and free ICG at an ICG dose of 1.0 mg/kg. Red solid line, popliteal (PO) LN. The fluorescence signals were acquired at 840 nm with excitation at 745 nm and were measured in radiance per cm^2^ per second per steradian (p/s/cm^2^/sr). (B) Quantitative analysis of the fluorescence signal of PO LN at different time points. (C) NIR fluorescence images of the isolated major organs and PO LN at 24 h post-injection with IM or ITM or free ICG. (**P<0.01)

Free ICG has already been used in clinical diagnostic applications, including SLN navigation during surgery. To test whether ITM was suitable for SLN imaging, ITM and IM were compared with free ICG after intradermal injection into the right paws of normal BALB/c nude mice. No obvious differences in the flow of the contrast agents through the popliteal (PO) LN were observed in the initial 5 min after injection, despite the stronger fluorescence signal of ITM and IM, indicating that the ICG-loaded micelles did not flow at a slower rate than the free ICG. The NIR fluorescence signal of these two micelles was maintained for 12 h. The NIR fluorescence signal of free ICG in SLN decreased quickly within 1 h due to loss of fluorescence after binding to nonspecific plasma proteins. At 30 min, one major difference was observed between free ICG and ICG-loaded micelles: secondary draining LNs were visualized after ITM or IM injection but not with free ICG. These LNs might be much deeper compared with the PO LN. The penetrating ability of ICG-loaded micelles was increased compared with free ICG (Figure 5A). Quantitatively, there was no significant difference in the imaging of normal PO LN between ITM and IM at the same time point. However, a stronger fluorescence signal was observed in the PO LN of normal mice injected with ICG-loaded micelles compared with the free ICG at all time points (Figure 5B). *Ex vivo* NIR images were obtained of the normal PO LN dissected from the above mice at 24 h (Figure 5C), confirming* in vivo* imaging results. The majority of ICG was accumulated in the liver, with lower amounts of ICG recovered from the spleen and kidney. These results indicated that ITM and IM with a diameter of 100 nm were useful for LN mapping, which could quickly move from the injection site to the SLN along lymphatic capillaries and remain in the SLN. Moreover, there was no significant difference in the imaging of normal LNs between these two micelles at the same time point, which suggested that these micelles accumulated in normal LNs by passive targeting.

### Optical imaging of tumor metastatic SLN and normal SLN in a HeLa tumor metastatic model

**Figure 6:**
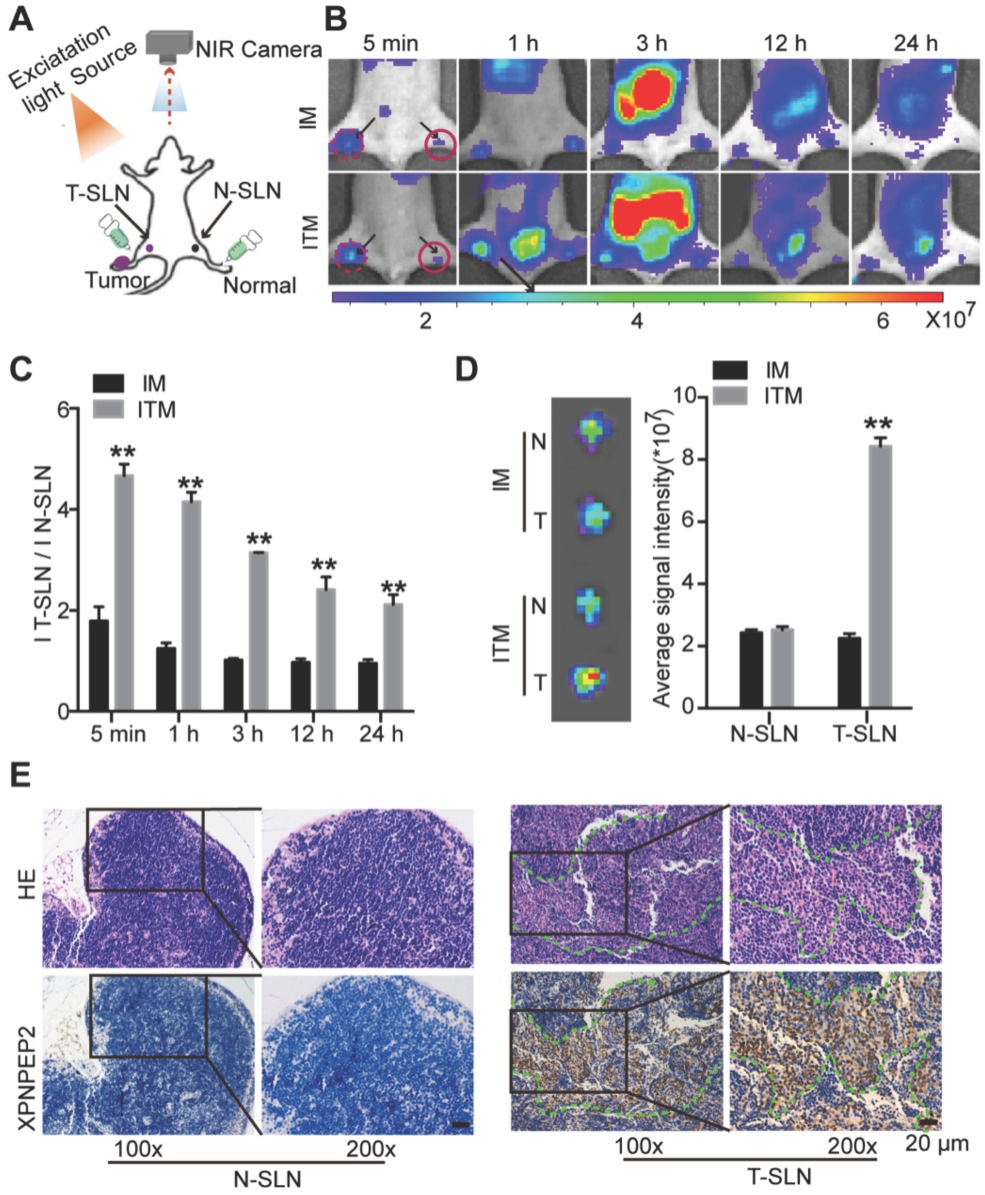
NIR fluorescence imaging of bilateral sentinel lymph nodes (SLNs) in HeLa tumor metastatic mice. (A) Schematic of bilateral SLNs fluorescence optical imaging. Right is the tumor metastatic SLN (T-SLN). Left is the normal SLN (N-SLN). (B) NIR fluorescence images of T-SLN and N-SLN hockinjected with IM or ITM (ICG does, 2 μg) at different time points (5 min, 1 h, 3 h, 12 h and 24 h); dotted line, T-SLN; solid line, N-SLN. The fluorescence signals were acquired at 840 nm with excitation at 745 nm and were measured in radiance counts per cm^2^ per second per steradian (p/s/cm^2^/sr). (C) Quantitative analysis of the fluorescence signal ratio of T-SLN and N-SLN at different time points. (D) *Ex vivo* NIR fluorescence images and quantitative analysis of the fluorescence signals of N-SLN and T-SLN at 1 h post-injection. (E) H&E staining and XPNPEP2 expression of N-SLN and T-SLN (scale bar, 20 μm). (**P<0.01).

For most of SLN mapping agents, it is difficult to distinguish between tumor metastatic SLN (T-SLN) and normal SLN (N-SLN) based on the images. To compare ITM or IM imaging of T-SLN and N-SLN, the same ICG-loaded micelles were injected into the bilateral hind footpads of the right HeLa tumor PO LN metastasis model (Figure 6A). To develop the SLN tumor metastasis model, HeLa-luc cells were planted in the right footpad of mice. After 4 weeks, luciferase bioluminescence and H&E staining detected the formation of tumor metastases in PO LN (Figure S8). The fluorescence intensity of IM in T-SLN was slightly stronger than that of N-SLN just at 5 min post-injection, and then there was no significant statistical difference between T-SLN and N-SLN. The strong fluorescence signal displayed from T-SLN may be due to the tumor induced lymphangiogenesis, which promoted IM to delivery into T-SLN compared to N-SLN. And IM could not persist for a long time due to passive targeting. On the contrary, the fluorescence signal of ITM in T-SLN increased rapidly and reached its maximum within 1 h, while the fluorescence signal of N-SLN was significantly weaker and increased less than T-SLN within 1 h of mice injection of ITM (Figure 6B). More obviously, at all the time points imaged, T-SLN displayed stronger fluorescence signals than N-SLN after the injection of ITM. ITM resulted in 4.73-fold, 4.12-fold and 3.34-fold higher accumulation in T-SLN compared to N-SLNs respectively at 5 min, 1 h and 3 h post injection (Figure 6C). SLNs on both sides were acquired after injection of IM or ITM for 1 h. *Ex vivo* imaging confirmed the more accumulation of ITM in T-SLN, which was similar to *in vivo* results (Figure 6D). Together, these results suggested that the ligand effects of TMTP1 promoted micelles rapidly accumulation in HeLa cell metastatic SLN, which could be clearly distinguished from the gradual accumulation in N-SLN, as significantly shown by the ITM accumulation ratios of T-SLN vs. N-SLN (Figure 6C-D). It was supposed that ITM could arrive in the tumor via interaction with XPNPEP2 expressed on the tumor cells. Thus, to shed light on the mechanisms of targeting accumulation, we detected the distribution of XPNPEP2 in above T-SLN and N-SLN. The staining of XPNPEP2 revealed the specific expression in T-SLN, while it was not present in N-SLN (Figure 6E). H&E staining also supported the tumor cell metastasis in the T-SLN. Thus, such high availability of XPNPEP2 expression on the tumor cells facilitated ITM active targeting of tumor cells.

### Optical imaging of tumor metastatic SLN and draining LNs in a HeLa tumor metastatic model

**Figure 7:**
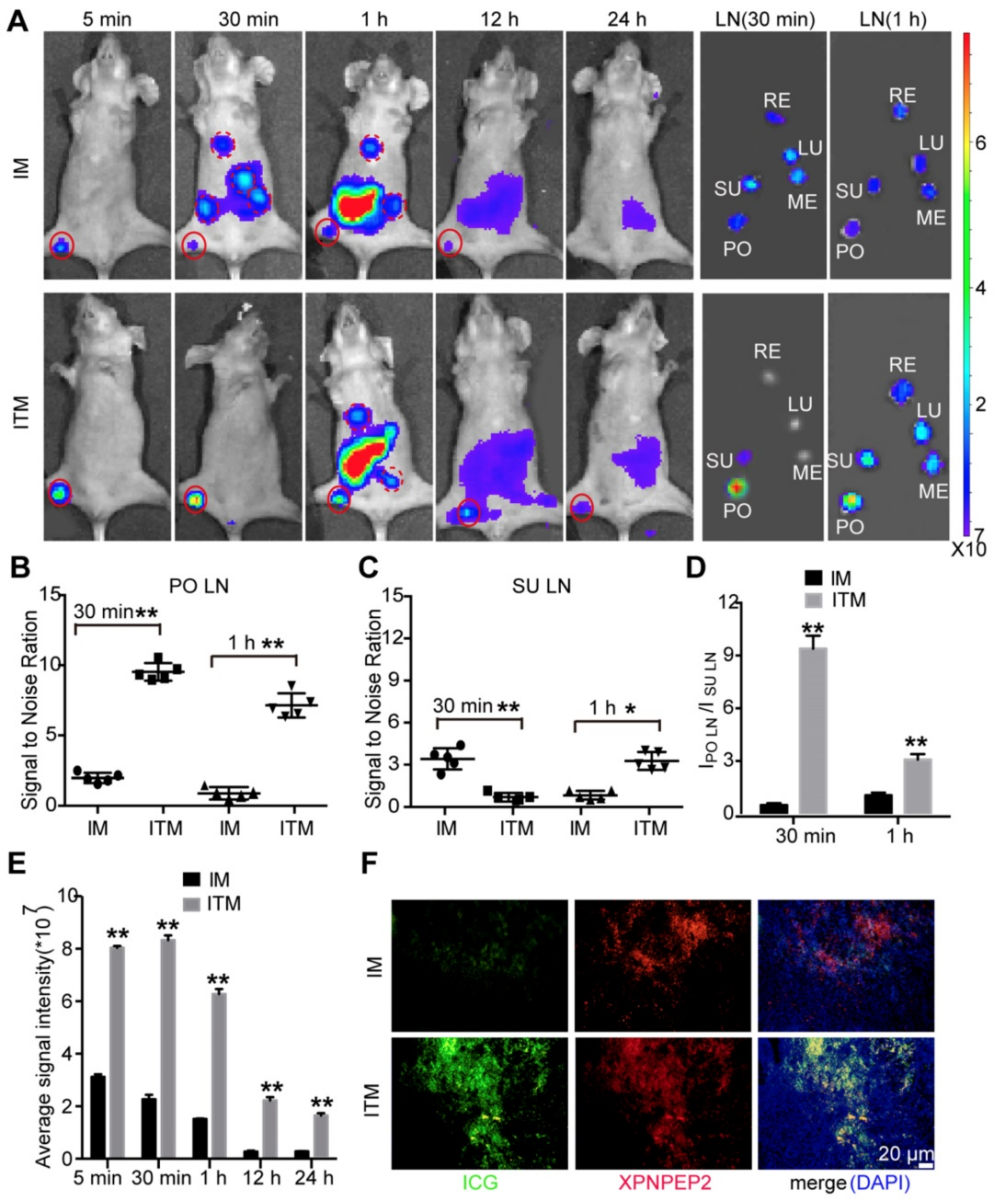
*In vivo* NIR fluorescence SLN imaging of tumor metastasis mice. (A) NIR fluorescence images of T-SLN and draining LNs after injection of IM or ITM (ICG dose, 2 μg) at different time points (5 min, 30 min, 1 h, 12 h and 24 h) and *ex vivo* NIR fluorescence images of dissected LNs at 30 min and 1 h postinjection (PO, popliteal; SU, subiliac; RE, renal; LU, lumbar aortic; ME, medial iliac). The red solid line represents PO LN. The red dotted line represents draining LNs. The fluorescence signals were acquired at 840 nm with excitation at 745 nm and were measured in radiance counts per cm^2^ per second per steradian (p/s/cm^2^/sr). (B) Scatter plots of *ex vivo* signal-to-noise ratio values for PO LN and (C) SU LN at 30 min and 1 h post-injection of IM or ITM. (D) Quantitative analysis of the fluorescence signal ratio of PO LN and SU LN at 30 min and 1 h. (E) Quantitative analysis of the fluorescence signals of T-SLN at different time points. (F) Confocal fluorescence images of the colocalization of ICG-loaded micelles (green) and XPNPEP2 (red) in T-SLNs (scale bar, 20 μm). The fluorescence images were obtained at excitation wavelengths of 405 nm, 633 nm and 561 nm for DAPI, ICG and XPNPEP2, respectively. Data are expressed as the mean ± s.e.m. (*P<0.05, **P<0.01).

An ideal SLN imaging agent should target the tumor metastatic SLN and remain in the SLN for a long time for the operator to effectively distinguish SLN from secondary draining LNs. To compare ITM or IM imaging of tumor metastatic SLN and draining LNs, ITM or IM were intradermally injected into the right hind footpads of the SLN tumor metastasis model as in Figure S8, and the migration of these two ICG-loaded micelles was recorded by NIR. As shown in Figure 7A, SLN emitted remarkably bright fluorescence at 5 min after injection of ITM and maintained high fluorescence for 12 h. The SLN in the IM group also showed fluorescence for a short time after being injected with IM, but the intensity of the fluorescence was lower than that observed in the SLN of mice injected with the ITM. Draining LNs, such as the subiliac lymph node (SU LN), were not visible at 5 min post-injection with these two ICG-loaded micelles. However, IM rapidly accumulated in the SU LN at 30 min post-injection, and the fluorescence signal of the draining LNs was stronger or similar compared with the PO LN, which illustrated that IM was not specific for imaging of metastatic LN. However, compared with the draining LNs, PO LN of the mice injected with ITM displayed a stronger fluorescence signal and were easily recognized.

The fluorescent LNs were excised for *ex vivo* NIR imaging at 30 min and 1 h post-injection, and the results were consistent with the *in vivo* results (Figure 7A-C). The ratio of PO LN to SU LN of mice who received ITM were 10.12 at 30 min and 3.23 at 1 h, while the ratio of PO LN to SU LN of mice that received IM were 0.67 at 30 min and 1.16 at 1 h (Figure 7D). These results demonstrated that ITM could remain in the metastatic SLN for a long time and was appropriate for SLN mapping. At 1 h post-injection of ITM, metastatic SLN could be easily distinguished from draining LNs. Quantitatively, a stronger fluorescence signal was observed in the tumor metastatic SLN injected with ITM compared with IM at all time points examined. ITM resulted in even 4.21-fold higher accumulation in tumor metastatic SLN compared with IM at 30 min post-injection (Figure 7E). Finally, confocal imaging of SLNs confirmed the high accumulation of ITM in the metastatic SLN compared to the IM group, and ITM mostly co-localized with XPNPEP2 expression on tumor cells in metastatic SLN (Figure 7F and Figure S9A). H&E staining supported the tumor metastasis in the above SLNs (Figure S9B). These results showed that ITM was useful for SLN mapping, which could actively target metastatic SLN and retain for a long duration to supply an adequate operation time. The nearly 1-h interval was sufficient for the surgeon to perform SLN biopsy and pathological examination to obtain sufficient information to determine the degree of surgical intervention.

### The imaging of ITM in K14-HPV16 transgenic mice

**Figure 8:**
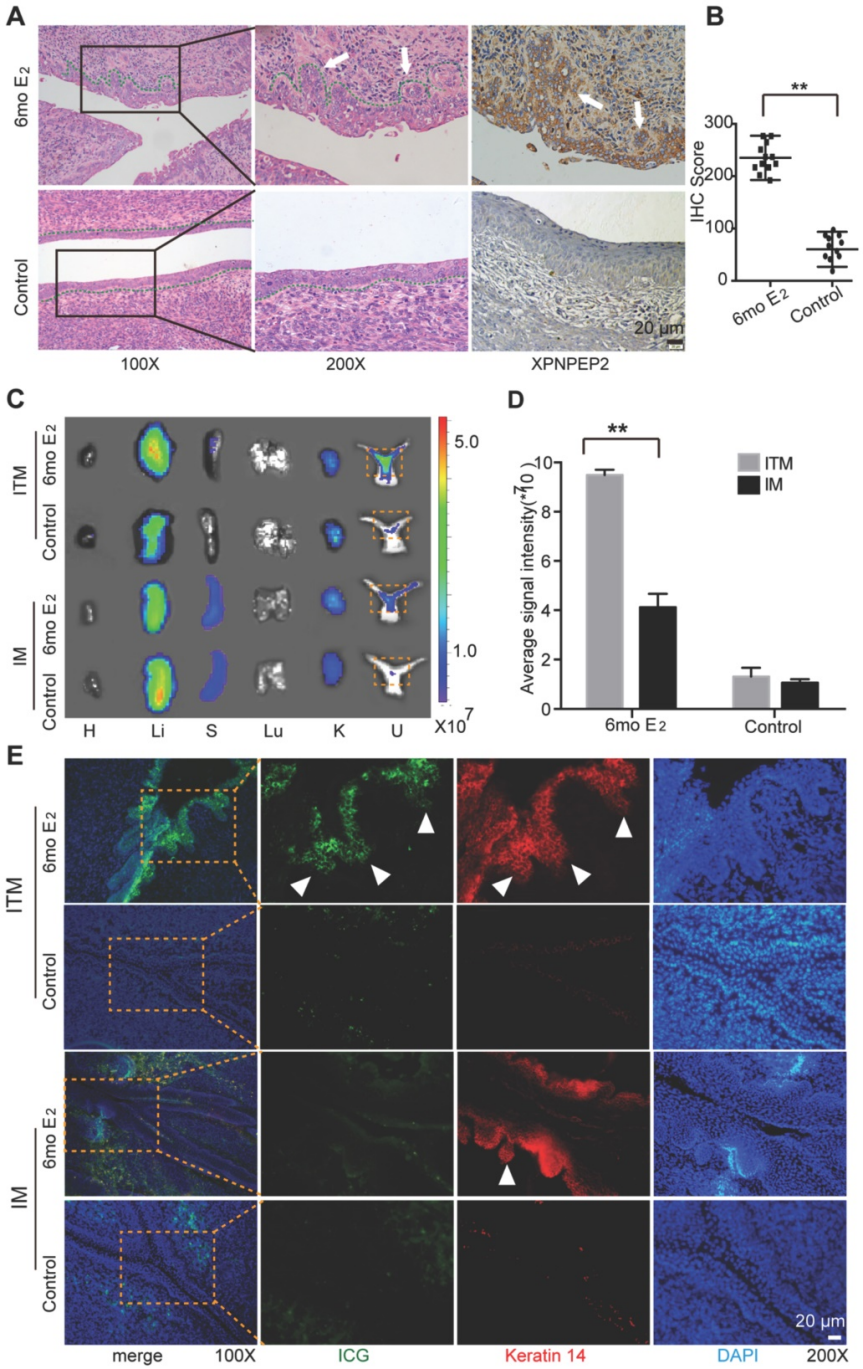
The imaging of cervical cancer in a K14-HPV16 transgenic mode. (A) Shown were representative images of H&E staining and XPNPEP2 expression of cervical epithelium from indicated groups (n=12). 6mo E2 group was that K14-HPV16 transgenic mice were treated for 6 months with a slow-releasing 17 -estradiol (E2) tablet (0.05 mg, 60 days). The control group was the nontransgenic mice treated with E2 for 6 months. (B) The IHC score of XPNPEP2 expression in cervical intraepithelial tissues of indicated groups. (C) NIR fluorescence images of the isolated major organs and uterus 24 h post-injection with IM or ITM at a ICG dose of 1.0 mg /kg in 6mo E2 group and control group. dotted line, cervix. (H, heart; Li, liver; S, spleen; Lu, lung; K, kidney; U, uterus). The fluorescence signals were acquired at 840 nm with excitation at 745 nm and were measured in radiance counts per cm^2^ per second per steradian (p/s/cm^2^/sr). (D) Average ICG fluorescence intensities of cervix from 6mo E2 group and control group. (E) Confocal fluorescence images of co-localization of Keratin 14 (red) and ICG (green) in cervix tissues. Nuclei was stained as blue with DAPI. The fluorescence images were obtained at excitation wavelengths of 405 nm, 633 nm and 561nm for DAPI, ICG and Keratin 14 respectively. (scale bar, 20 μm). White triangles represent micrometastases that break through the basement membrane. (**P<0.01).

To further demonstrate that ITM micelles could target CC, K14 double-transgenic mice were used, which expressed both the HPV16 E6 and E7 oncogenes. After treatment with exogenous estrogen (E_2_) at a physiological level for six months, the K14-HPV16 transgenic mice developed CC with high penetrance 26. Histological analyses of the mouse cervical canal showed that six-month treatment with E_2_ (6 mo E_2_ group) resulted in CC in all mice, which was consistent with other previously published results. The control group consisted of the nontransgenic mice also treated with E_2_ for six months, which could not develop CC (Figure 8A) 27. Moreover, immunohistochemistry (IHC) analysis of XPNPEP2 expression in cervical squamous epithelium showed an incremental increase in XPNPEP2 expression within cancerous lesions of the squamous epithelium in 6 mo E_2_ group mice, whereas XPNPEP2 expression was almost negative in cervical sections of control mice (Figure 8A-B). Therefore, 6 mo E_2_ group and control mice were used to administer an intravenous injection of ITM to detect whether ITM could target primary cancers of CC, especially microinvasive carcinoma. At 24 h post-injection, the animals were sacrificed, and their organs (heart, liver, spleen, lung, kidney and uterus) were exposed for *ex vivo* NIR fluorescence imaging to evaluate the localization of ITM and IM (Figure 8C). ITM resulted in 3.45-fold higher accumulation in the cervical tissues of CC compared with IM. However, for control mice, no difference was observed between the micelles (Figure 8D). To clarify the location of micelles in the cervix, the cervixes of the above mentioned mice were sectioned. The morphology of the mouse cervical canal in frozen sections was shown in Figure S10A. ITM was accumulated within the multiple layers of the cervix epithelium in 6 mo E_2_ group mice, especially in the microinvasive carcinoma breaking through the basement membrane (Figure 8E). In contrast, littler fluorescence of ICG was observed in the 6 mo E_2_ group injected with IM micelles. In the control mice, there was almost no fluorescence of either micelle. Quantitative analysis of ICG fluorescence intensity in the cervix epithelium was performed using ImageJ software (Figure S10B). Moreover, ITM colocalized with Keratin 14 in the cervix epithelium in 6 mo E_2_ group mice, which was diffusely positive in invasive squamous cell carcinoma 28. Our results showed that ITM could actively accumulate in cancerous lesions of the cervical epithelium, especially in epithelium microinvasive carcinoma to interstitial sites, which suggested that TMTP1 could not only target primary cancers of cervical cancer but also the metastases.

### *In vivo* Toxicity of ITM

To examine the toxicity of micelles *in vivo*, high doses of IM or ITM were intravenously injected into healthy mice (Figure S11). There were no significant changes in body weight or in physical features over the 28 days post-injection. The mice were sacrificed at 28 days post-injection. Histological analysis of major organs showed that no signs of overt toxicity such as tissue degeneration or necrosis, compared to the effects of PBS on the treated mice. The levels of ALT, AST, and BUN in nude mice in the IM and ITM groups were normal compared with the nude mice in the PBS group. These results strongly suggested that the ICG-loaded micelle formulation was nontoxic *in vivo* at the dosage of 10 mg/kg, which is approximately 10 times higher than the imaging dose of ICG used in most studies. To further demonstrate the safety of micelles, we performed a repeat-dose toxicity study. Three different doses (1 mg/kg, 3 mg/kg, 5 mg/kg) of ITM were intravenously injected into mice every day over one month. There were no significant changes in body weight or in physical features during this period. Histological analysis of major organs after the mice were sacrificed also showed no signs of overt toxicity compared with the effects of PBS on the treated mice (Figure S12). Our results confirmed the nontoxicity and biocompatibility of these two ICG-loaded micelles.

## Discussion

Most patients with early cervical cancer are treated by radical hysterectomy and pelvic lymph node dissection, which carries a high risk of postoperative complications 29. Current medical practices focus on minimizing mortality and improving quality of life. Evidence suggests that SLN biopsy and nonradical surgery are safe approaches for the staging and management of early cervical cancer to significantly reduce treatment-related morbidity 30. Therefore, the diagnosis of cervical cancer lesions, especially metastasis, is critical for optimization of surgical resection. In this study, we describe a noninvasive and intraoperative method to sensitively identify tumor metastases and SLN imaging, particularly T-SLNs, using ICG-loaded micelles modified with the tumor metastasis peptide TMTP1.

Squamous cell carcinoma and adenocarcinoma are the most common histological subtypes of cervical cancer. The previous literature describes high expression of XPNPEP2 in cervical cancer, and 80% of cervical cancer (25/30) patients have a XPNPEP2 histological score of more than 150 (the total histological score is 300) 19. To further determine the expression of XPNPEP2 in cervical cancer, we added 45 cervical cancer specimens in this study. The results showed that more than 80% of XPNPEP2 scores exceeded 150, and the expression of XPNPEP2 in micrometastases was strongly positive (Figure S13). We combined a fluorescent imaging agent and the novel tumor metastasis targeting peptide TMTP1, the potential receptor of which, XPNPEP2, was significantly upregulated in cervical cancer tissues, especially in micrometastases, thus providing the possibility of the wide use of ITM for clinical imaging of situ and cervical cancer metastasis.

To achieve a negative tumor margin and ensure minimal surgical complications, it is important to accurately determine the size and extent of the cancer lesions, especially metastases, during surgery. Both CT and MRI are not sufficiently sensitive to detect metastases 31. We demonstrated that ITM micelles could accumulate in primary cervical cancer and metastatic foci by active targeting and achieve good tumor localization *in vivo*. Endoscopic and robot-assisted laparoscopic surgery as well as fluorescence-guided surgery are receiving increasing attention. They can reduce the incidence of surgery-related morbidity and allow more precise resection. Maximizing the implementation of cytoreductive surgery and minimizing tumor residuals might increase overall survival. The wide application in the clinic of the da Vinci Si robot platform and fluorescent laparoscopic techniques provide more possibilities for the use of near-infrared fluorescent imaging agents in surgery 32-34. In the future, ITM micelles are expected to achieve clinical transformation for intraoperative guidance of cervical cancer-related surgeries, aiming to provide a real-time and accurate image of metastasis.

For patients with early-stage cervical cancer, LN metastasis has been proven to be a critical risk factor related to survival 35. However, LN dissection may lead to lymphedema of the legs, nerve damage and lymphocyst formation in 14-32% of patients. To reduce such surgical complications, the concept of SLN imaging has been gradually introduced and applied 36. In our study, ITM micelles with a diameter of 100 nm could quickly move from the injection site to SLNs along lymphatic capillaries and remain in the SLNs for a long time, which is in line with the requirements of an ideal contrast agent 37, 38. However, most lymphatic mapping agents are incapable of diagnosing SLN metastases or are not visible on the images 39, 40. Thus, there is future scope to remove the reliance on biopsy to determine the tumor burden of a patient, with the implementation of highly specific tumor-targeted contrast agents. ITM micelles could actively target metastatic SLNs and remain in the SLNs for a long time without flowing to secondary lymph nodes. Thus, SLNs cannot only be easily recognized from draining LNs but also be identified whether with tumor metastasis or not. Therefore, ITM may provide accurate and real-time intraoperative guidance, completely eliminating the time spent waiting on biopsy results. In addition to real-time NIR fluorescence imaging, afterglow optical agents, which emit light long after cessation of excitation, hold promise for ultrasensitive imaging* in vivo* and a few inorganic nanoparticles have been shown to produce afterglow in biologically relevant conditions^41^, ^42^. In the future work we will try to apply the tumor metastasis targeting peptide in the afterglow imaging for tumor diagnosis.

Due to the increasing opportunities in the surgical field, more fluorescence imaging systems are becoming available for both open and laparoscopic surgery. To date, ICG and methylene blue are the o fluorophores that are approved for clinical use by the FDA. Rossi et al. used ICG fluorescence imaging with the da Vinci® robot for laparoscopic surgery in patients with cervical or endometrial cancer 33. SLNs were successfully identified in 17 of 20 patients (85%). However, ICG is nonspecific and their chemical structures do not allow conjugation to tumor-specific ligands. Therefore, it is mainly suitable for indications such as SLN mapping, since they do not bind to tumors but only follow the lymphatic drainage pattern. Thus, in our study, we applied nanocarriers to overcome the nontargeting of free ICG. Compared to free ICG, ITM deposit in blood (5-10 times), deep-tissue molecular optical imaging, good photostability and targeting of tumor metastasis. The ITM could not only target lymph nodes but also tumor metastatic tissues, demonstrating the potential to identify tumor cells that are invisible to the naked eye in surgery. To determine whether the targeted accumulation in the tumor is determined by adjusting the length of the injection, in the subsequent experiments, we will increase the imaging of tumor metastasis using different doses at different time points, thus providing reliable parameters for subsequent clinical applications.

Recently, NIR-induced photodynamic therapy (PDT) and photothermal therapy (PTT) have been extensively developed to optimize the effects on tumor ablation 43, 44. Nanocarriers can simultaneously load imaging agents and therapeutic drugs to achieve integration of diagnosis and treatment 45, 46. Imaging-guided PDT and PTT can provide individualized and precise treatment for cancer patients. ICG as a diagnostic and photodynamic agent, one of the major limitations is its instability in aqueous solution, which decreases the efficacy of its PTT. We have demonstrated that ITM could effectively inhibit the growth of tumors in nude mice under laser irradiation, reflecting the enormous potential of TMTP1-modified PEG-PLGA micelles as delivery systems for ICG for its use in tumor-diagnosis and therapy. In the next article we will elaborate on the PTT and PDT effects of ITM in cancer therapy.

K14-HPV16 transgenic mice develop epidermal hyperplastic lesions that progress to dysplastic lesions and ultimately to invasive cancer, which is in line with the occurrence and progress of clinical cervical cancer 47, 48. Our results showed that ITM micelles could actively accumulate in cancerous lesions of the cervical epithelium, especially in the microinvasive carcinoma of the interstitial site. Our previous study has suggested that XPNPEP2, the potential receptor of TMTP1, can serve as a potential biomarker in metastatic cervical cancer. TMTP1 has a remarkable ability to target the very early stage of occult metastatic foci 15. Thus, we further demonstrated that ITM micelles could not only target primary cancers of cervical cancer but also occult metastases in a human papillomavirus-transgenic mouse model. Recently, colposcopy with biopsy has been shown to link screening and diagnosis/treatment of cancerous lesions but is diagnostically poor and subjective. The need to improve colposcopy is widely recognized. In this study, ITM micelles could mainly accumulate in the cancerous lesions of the cervical epithelium and offer a potential approach to increase sensitivity and specificity of early cervical cancer detection.

Although the preclinical results are very promising, the perceived acute and chronic toxicity of probes, such as organic dyes, quantum dots, upconversion nanoparticles, metal nanoclusters, carbon-based, and silica-based nanomaterials, may impede clinical translation 11, 49. Thus, in this study, we chose PEG-PLGA polymeric micelles as the nanocarrier of ICG. PEG and PLGA are completely biocompatible and biodegradable, and they have already been approved by the FDA 20. PLGA is one of the best characterized biodegradable copolymers, which decomposes to nontoxic products (H2O and CO2) that are eliminated from the body. Surface modification with PEG increases the hydrophilicity of the formulation to yield a stealth particle with an enhanced blood circulation time and improved pharmacokinetics by preventing opsonization, as well as uptake by the mononuclear phagocyte system 50. Our ICG-loaded micelles were prepared using a nanoprecipitation method. The micelle synthesis process is simple and permits easy control of the conditions, which provides hope for conversion to clinical mass production in the future. A few polymeric micellar formulations have been used clinically, e.g., Genexol®-PM, a kind of paclitaxel-loaded PEO-PLA polymeric micelle, has already undergone phase IV clinical trials in patients with taxane-pretreated recurrent breast cancer 51. In our study, we confirmed the nontoxicity and biocompatibility of ICG-loaded micelle *in vivo*, which provided laboratory data for future clinical transformation.

In conclusion, by combining the novel tumor metastasis targeting polypeptide TMTP1 and NIR agents ICG, the NIR optical imaging can map the primary tumor, metastases and SLNs. The easy preparation, excellent NIR SLN imaging quality, and biosafety suggest that ITM micelles have great potential for clinical translation to map SLNs and provide intraoperative guidance. Importantly, TMTP1-modified ICG-loaded micelles can identify metastatic SLNs, which are prime targets for the efficient treatment of cancer metastasis. Therefore, the TMTP1-modified micelles system could provide an effective drug delivery platform with efficient lymph metastasis targeting ability to improve therapeutic outcomes for cancer metastasis. Moreover, our studies confirm the viability of TMTP1-modified micelles in the clinical diagnosis of cancerous lesions and metastatic cervical cancers. We hope that our research will provide new strategies for diagnosis and therapy of tumor metastases as well as the development and clinical translation of nanomedicine.

## Materials and Methods

### Synthesis of ICG-loaded PEG-PLGA micelles modified with or without TMTP1

HCL.NH2-PEG-NH2.HCL (Mw 3500) was purchased from Beijing Kaizheng Bioengineering Corporation (Beijing, China). PLGA-COOH (Mw10000) was obtained from Shandong Medical Device Research Institute (Jinan, China). MPEG5000-PLGA50/50 was purchased from Jinan Daigang Biomaterial Co., Ltd (Jinan, China). Dichloroethane (EDC), N-hydroxysuccinimide (NHS), maleimide (MA) and ICG were purchased from Sigma-Aldrich (MO, USA) and used without further purification. N, N-dimethylformamide (DMF), dichloromethane (DCM), diethyl ether and methanol were purchased from Aladdin Reagent (Shanghai China). The cyclic polypeptide TMTP1 (NVVRQC, end-collateral amide bond into a ring) was synthesized in Wuhan Baiyixin Biotechnology Co., Ltd (Wuhan Chian).

MA was dissolved in DCM and was activated by EDC and NHS for 5 h. Then, the proper amounts of HCL.NH2-PEG-NH2.HCL and triethylamine were added into the above solution for further reaction for 48 h to obtain MA modified PEG solution. Secondly, EDC and NHS were used to activate the PLGA-COOH in DCM for 5 h to obtain the activated PLGA, which subsequently was added into the above MA modified PEG solution for another 48 h to synthesize the MA modified PEG-PLGA. Finally, the above crude products were dropwise added into the mixture solution (ratio of diethyl ether/methanol was 5/1), the precipitation was washed by the mixture solution twice to purify the products and the final Mal-PEG-PLGA were dried in vacuum for 12 h. TMTP1 polypeptide and MA modified PEG-PLGA (1:20, w/w) were dissolved in 1mL DMF solution, stirring continuously at room temperature for 8 h to obtain PLGA-PEG-TMTP1.The ICG-loaded PEG-PLGA micelles were prepared by a one-step self-assemble method. 200 μg free ICG were added into the above 1 mL DMF solution of PLGA-PEG-TMTP1 (20 mg/mL). This mixture was added dropwise under stirring into 15 mL of deionized water. After 30 min, the above solution was centrifuged for 30 min at 13,000 rpm to collect the micelles and then washed with water twice. The final micelles were re-dispersed under sonication in 1 mL water. The ICG-NP micelles were prepared in a similar way except that PLGA-PEG-TMTP1 was replaced by PEG-PLGA copolymer.

### Physico-chemical characterization of micelles

The efficiency of the click chemistry between the peptide and MA-PEG-PLGA was determined. A series of known concentrations of free polypeptides were detected by HPLC. TMTP1 polypeptide and MA modified PEG-PLGA (mole ratio1:1) were dissolved in 1mL DMF solution, stirring continuously at room temperature for 8 h to obtain PLGA-PEG-TMTP1.Then the HPLC was used to measure the free polypeptide in the above solution that was not reacted with MA-PEG-PLGA.

The medium diameter, size distribution and zeta potential of ICG-loaded micelles were detected by DLS (Zeta Plus, Brookhaven Instruments, USA). The structure and morphology of ICG-loaded micelles were determined by TEM (JEM-1230, Japan). The encapsulation efficiencies (EE) of ICG of both the two ICG-loaded micelles were determined. The micelles after precipitating were isolated from the aqueous medium by ultracentrifugation (13,000 r/min, 30 min). UV/vis spectrometer was used to measure the free ICG of the supernatant at λmax of 784 nm. The EE of ICG-loaded micelles were calculated using the formula EE (%) = (ICGTotal - ICGFree) / ICGTotal × 100%.

### *In Vitro* Release Profile Study

To study the release profile of ICG-loaded micelles *in vitro*, 3 mL ITM or IM aqueous solution (containing 30 μg/mL ICG) was infused into a dialysis tube (M.W. 3500 Da) and dialyzed against phosphate-buffered saline (PBS, pH = 7.4) containing 10% FBS at 37 °C under continuous shaking in the dark. At each predetermined time point over a period of 180 h, the ICG concentration in the dialysate was measured by fluorescence spectrophotometer (F-4600; Shimadzu, Tokyo, Japan) with excitation and emission wavelengths of 745 nm and 840 nm. The total volume of dialysis medium was maintained at 50 mL through the test. Experiments were performed three times.

### Photostability of micelles

The effect of the light exposure on the degradation of ITM or IM was determined with visible light at room temperature. The ICG and ICG-loaded micelles were diluted with distilled water to a final concentration of 10 μg/mL and were loaded into a 12-well plate. The fluorescence intensity was measured for up to 6 days. After incubation, the remaining fluorescence of each sample was measured using a spectrofluorometer with excitation and emission wavelengths of 745 nm and 840 nm, respectively. For the quantitative analysis, we normalized the fluorescence signal intensity.

### Cells and Animal

The cervical cancer cell lines C-33A, SiHa, and HeLa and the human cervical epithelial cell line End1 were purchased from the American Type Culture Collection (ATCC, Manassas, VA, USA). All cervical cancer cells were cultured in Dulbecco's modified Eagle's medium (DMEM) supplemented with 10% fetal bovine serum (FBS), 100 IU/mL penicillin, and 100 μg/mL streptomycin (Invitrogen). End1 cells were cultured in keratinocyte serum-free medium supplemented with 0.1 ng/mL human recombinant epithelial growth factor, 0.05 mg/mL bovine pituitary extract, and 44.1mg/L (final concentration 0.4 mM) calcium chloride. The immortalized human cervical keratinocyte cell line S12 was a gift from Kenneth Raj and were cultured according the previous literature.

The female 4 -week-old BALB/c-nude mice were purchased from Beijing HFK Bioscience Co. Ltd. All animal procedures were approved by the Ethics Committee for Animal Experiments of Hubei province. The K14-HPV16 transgenic mice were gifted by the National Cancer Institute Mouse Repository (Frederick, Maryland, USA) and bred at the Experimental Animal Center, HUST as before.

### Cell Viability

For each CCK-8 assay, End1, SiHa, HeLa, S12 and C-33A cells were seeded in 96 well plates. The culture medium was replaced by 100 μL serum-free culture medium containing different concentrations (0, 1, 10, 20, 40 and 80 μg/mL) of ITM or IM for 24 h. Then the cells were washed three times with PBS and 90 μL fresh medium and 10 μL CCK-8 solution (Dojindo Molecular Technologies, Japan) were added into the cells for 2.5 h. Then the optical density (OD) was measured with a Microplate Reader (Bio-Rad, USA) at 450 nm.

### *In Vitro* Cellular Uptake

The cervical cells (2 × 10^5^ cells/well) were seeded in 12-well plate glass coverslips overnight. Then the cells were treated with serum free medium with 10 μg/mL of ICG-loaded micelles for 3 h. After washing three times with PBS, the cells were stained by 4,6-diamidino-2-phenylindole (DAPI) with 1 μg/mL for 10 min at room temperature. For the competition assay on cervical cells, cells were pretreated with excess TMTP1 pepetide for 30 min and then incubated with 10 μg/mL ITM. The fluorescent images of the cells were acquired on a confocal laser scanning microscope (Zeiss LSM510, Goettingen, Germany). The fluorescence images were obtained at excitation wavelengths of 405 and 633 nm for DAPI and ICG, respectively. The NIR fluorescence images *in vitro* were obtained by IVIS Spectrum Imaging System (PerkinElmer, USA) with an excitation wavelength of 745 nm and an emission wavelength of 840 nm. For quantitative analysis, the cervical cells in 24-well culture plates were incubated with ICG-loaded micelles at an ICG concentration of 10 μg/mL for 3 h. After washing three times with cold PBS, cells were collected by flow cytometry on a FACS Calibur (BD Accuri C6).

### Immunohistology

Tumors, LNs and cervixes of mice were isolated and sectioned for IHC staining. The tissue slides were incubated overnight at 4 °C with the primary antibody for rabbit anti-XPNPEP2 (GTX109995, 1: 200; GeneTex, Irvine, CA, USA). The typical images were imaged with an Olympus BX53 microscope (Olympus, Tokyo, Japan).) For immunofluorescence, fresh tumors, LNs and cervixes were sectioned into 6 μm slices. After Fixing with 4% paraformaldehyde and blocking with PBS with 5% BSA, slices were incubated with antibody of rabbit anti-XPNPEP2 (1: 200) overnight at 4 °C and then Alexa Fluor 594-conjugated secondary antibody (1:200, Invitrogen). The slices were then incubated with medium containing DAPI and imaged with a confocal laser scanning microscope. The excitation wavelength of ICG was set at 633 nm and Alexa Fluor 594 was set at 561 nm.

### Pharmacokinetic study and Bio-distribution of ITM

Normal BALB/c-nude mice were used to determine the dynamics and bio-distribution of ITM. The mice were injected intravenously ITM and IM at equivalent ICG dose of 1.0 mg/kg. The NIR signals *in vivo* were imaged by IVIS Spectrum Imaging System (1 h, 2 h, 6 h,12 h, 24 h and 48 h) with an excitation wavelength of 745 nm and an emission wavelength of 840 nm. At different time point, 2 mice from each group were randomly sacrificed and the excised organs were imaged. The exact fluorescent radiant counts of the organs were measured with the region of interest (ROI) tool in the software.

Normal BALB/c-nude mice were administered intravenously with ITM or free ICG solution at an dose of 10 μg, respectively. At designed time points of 5 min, 10 min, 30 min, 1 h, 2 h, 3 h, 4 h, 8 h and 12 h post- injection, 0.5 mL blood samples were collected into EDTA tubes and centrifuged at 6000 rpm for 10 min to obtain plasma. A fluorescence spectrophotometer with excitation and emission wavelengths of 745 nm and 840 nm was used to measure the ICG of the supernatant. The ICG content in the plasma was then assayed.

### Animal models and *In vivo* and *Ex vivo* NIR imaging

For the cervical cancer subcutaneous tumor model, the cervical cancer cell line HeLa was transfected with a luciferase lentivirus (HeLa-luc). 10^7^ HeLa-luc cells were incubated into hips near the mouse spine. After 2 weeks, tumor was formulated about 150~200 mm^3^. Tumor volume (V) was calculated as follows: V = L × W2/2, where W is the width and L is length of tumor. HeLa-bearing tumor BALB/c-nude mice were divided into three groups randomly and injected intravenously ITM and IM at equivalent ICG dose of 1.0 mg/kg. Intravenous injection of 100 μL 100 μM TMTP1 before the administration of ITM was used as a competitive blocker. The NIR signals *in vivo* were imaged by IVIS Spectrum Imaging System (1 h, 2 h, 12 h, 24 h and 48 h) with an excitation wavelength of 745 nm and an emission wavelength of 840 nm. After 48 h injection, mice were sacrificed, then organs (heart, liver, spleen, lung, and kidney) and tumors were collected for* ex vivo* NIR imaging. Tumors were frozen and cut into 6 μm thick slices.

1× 10^6^ HeLa-Luc cells with expression of luciferase were injected into tail vein to establish lung metastasis mouse model. The formation of lung metastasis foci was examined weekly by bioluminescence imaging (BLI) (Caliper Life Sciences). Before imaging, mice were administrated with 150 mg/kg D-luciferin in 100 μL of saline via intraperitoneal injection and kept anesthetized by 5% isoflurane. Lung metastasis mice were divided into two groups randomly and injected intravenously ITM and IM at equivalent ICG dose of 1.0 mg/kg. After 24 h injection, mice were sacrificed, then lungs were isolated for NIR imaging.

For the SLN metastasis model, 1 × 10^7^ HeLa-luc cells in 15 μL PBS were injected into the right hock of 5-week-old BALB/c-nude mice. The mice were monitored by BLI weekly to detect tumor metastases. After 1 month, tumor was about 5 mm with PO LN metastasis. HeLa-bearing mice were divided two groups randomly (n=10) and injected ITM and IM micelles via foot at equivalent ICG dose of 2 μg. At designed time points (5 min, 30 min, 1 h, 12 h and 24 h), the NIR fluorescence images *in vivo* were obtained. At 30 min and 1 h post injection, 2 mice from each group were randomly sacrificed and the excised main LNs were imaged. After 24 h post injection, LNs and main organs (heart, liver, spleen, lung, and kidney) were handled as described. In the same way, normal mice were divided into two groups randomly (n=5) and injected intradermally ITM or IM via foot. The NIR fluorescence images were acquired as mentioned above.

Female progenies of K14-HPV16 transgenic mice were genotyped by PCR, and a slow-releasing 17 -estradiol (E_2_) tablet (0.05 mg, 60 days; Innovative Research of America) was inserted under the dorsal skin of both positive transgenic mice and nontransgenic mice every 2 months beginning at 4 - 6 weeks of age. After 6-month treatment with E_2_, the transgenic mice (6mo E_2_ group) and nontransgenic mice (Control group) were injected intravenously ITM and IM via tail vein at ICG dose of 1.0 mg/kg. After 24 h, we dissected the cervical tissue for NIR imaging *in vitro* and cut into paraffin sections and frozen sections for HE staining and immunofluorescence.

### Toxicity test of ITM *in vivo*

4-week-old female normal BALB/c-nude mice were divided into three groups (n=15), the ITM group, IM group and the blank group of PBS. The mice were subjected to tail vein injection of ITM and IM at 10 times of the ICG imaging doses (10 mg/kg) and the same volume of sterile PBS respectively. The mice were observed every two days, and the mice body weight was measured. At 28 days post injection, blood was taken from the eyeball, and the kits were used to test for the main biochemical indicators such as transaminase (ALT), aspartate aminotransferase (AST), blood urea nitrogen (BUN). The main organs (heart, liver, spleen, lung and kidney) were handled as described, fixed with 4% paraformaldehyde, paraffin sectioned, HE stained, inverted microscope observation and filming. For the repeat-dose toxicity study of ITM, 4-week-old female normal BALB/c-nude mice were divided into four groups (n=5), the low dose group (1 mg/kg), the medium dose group (3 mg/kg), the high dose group (5 mg/kg) and the blank group of PBS. The mice were subjected to tail vein injection of the corresponding dose of ITM and the same volume of sterile PBS respectively every day within one month. The mice body weight was measured every two days. After one month post injection, The main organs (heart, liver, spleen, lung and kidney) were handled as described, fixed with 4% paraformaldehyde, paraffin sectioned, HE stained, inverted microscope observation and filming.

### Statistical analysis

The results were analyzed by using unpaired Student's two-tailed t-test with GraphPad Prism 6.0 (GraphPad Software, CA, USA). Values of p < 0.05 were considered significant (*p < 0.05; **p < 0.01; ***p < 0.001; ****p < 0.0001). Each experiment was repeated at least three-times.

## Supplementary Material

Supplementary figures and tables.

## Figures and Tables

**Table 1:** The basic characterization of IM and ITM.

Micelles	Diameter (nm)	Zeta potential (mv)	Polydispersity	EE (%)
IM	98.3±5.4	-10.17±1.13	0.101	50±5.03
ITM	113.5±3.6	-13.25±1.41	0.198	52±8.14

**Table 2:** Area-under-the-curve (AUC, 0-12 h) in plasma after i.v. administration of 0.5 mg/kg ICG in ITM and free ICG.

Parameters	ITM	Free ICG
AUC, mg/L*min	146.39	13.458
MRT, min	265.57	268.17
t1/2, min	20.6	4
CL, L/min/kg	0.002	0.018
V, L/kg	1.996	21.358
T_max,_ min	5	5
C_max,_ mg/L	1.15	0.134

## Abbreviations

BLI: bioluminescence imaging
CC: cervical cancer
DAPI: 4,6-diamidino-2-phenylindole
DCM: dichloromethane
DMF: N, N-dimethylformamide
DLS: dynamic light scattering
EDC: Dichloroethane
EE: encapsulation efficiencies
E_2_: exogenous estrogen
FDA: Food and Drug Administration
HPLC: High Performance Liquid Chromatography
ICG: indocyanine green
IHC: immunohistochemistry
IM: ICG-loaded PEG-PLGA micelles
ITM: ICG-loaded TMTP1-PEG-PLGA micelles
LN: lymph node
MA: maleimide
MRI: Magnetic Resonance Imaging
NHS: N-hydroxysuccinimide
NIR: near-infrared
N-SLN: tumor metastatic sentinel lymph node
PDT: photodynamic therapy
PEG: poly (ethylene glycol)
PET-CT: Positron Emission Tomography-Computed Tomography
PLGA: poly (lactic-co-glycolic acid)
PTT: photothermal therapy
RES: reticulo-endothelial system
SLN: sentinel lymph node
TEM: transmission electron microscopy
T-SLN: tumor metastatic sentinel lymph node
ROI: region of interest
