# Supplementary material for: TMTP1-modified Indocyanine Green-loaded Polymeric Micelles for Targeted Imaging of Cervical Cancer and Metastasis Sentinel Lymph Node in vivo
Source: Theranostics. 2019 Sep 25;9(24):7325–44. doi: 10.7150/thno.35346 (PMC6831285; doi:10.7150/thno.35346)
Supplement: Supplementary file 1 — Supplementary figures and tables. [file thnov09p7325s1.pdf]

Supplementary

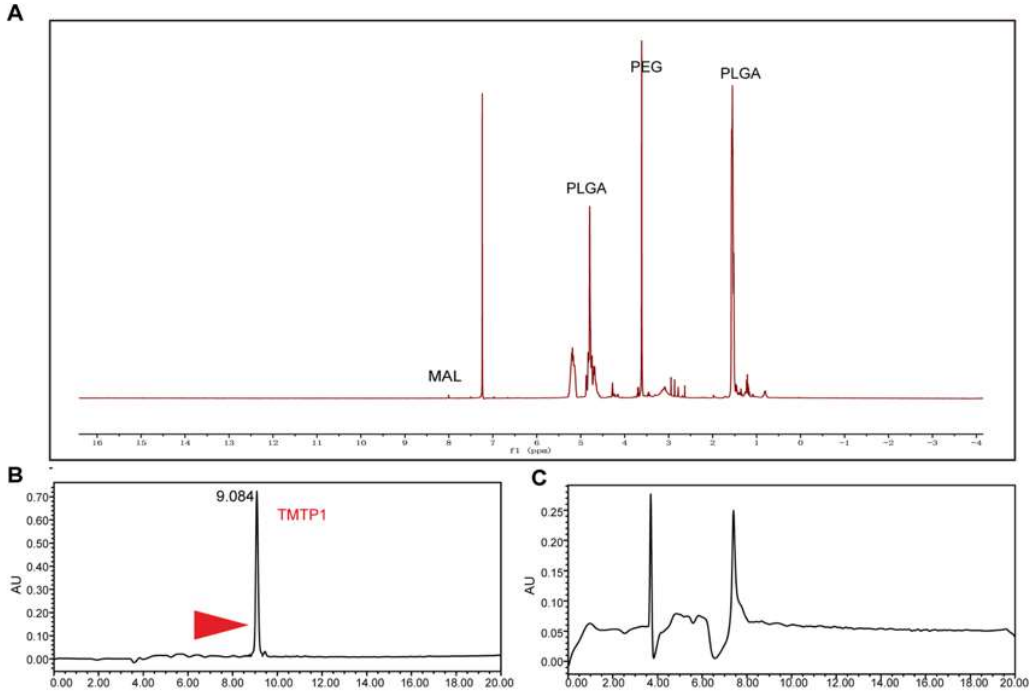

Figure S1: (A) The characterization by ^{1}H NMR spectrum of MA-PEG-PLGA. (B) HPLC detection of free peptide TMTP1. The peak of the TMTP1 peptide appears at 9.084 min. Red arrow is the peak. (C) HPLC detection of the supernatant of TMTP1 and PLGA-PEG-MAL polymer reacted at room temperature for 8 h.

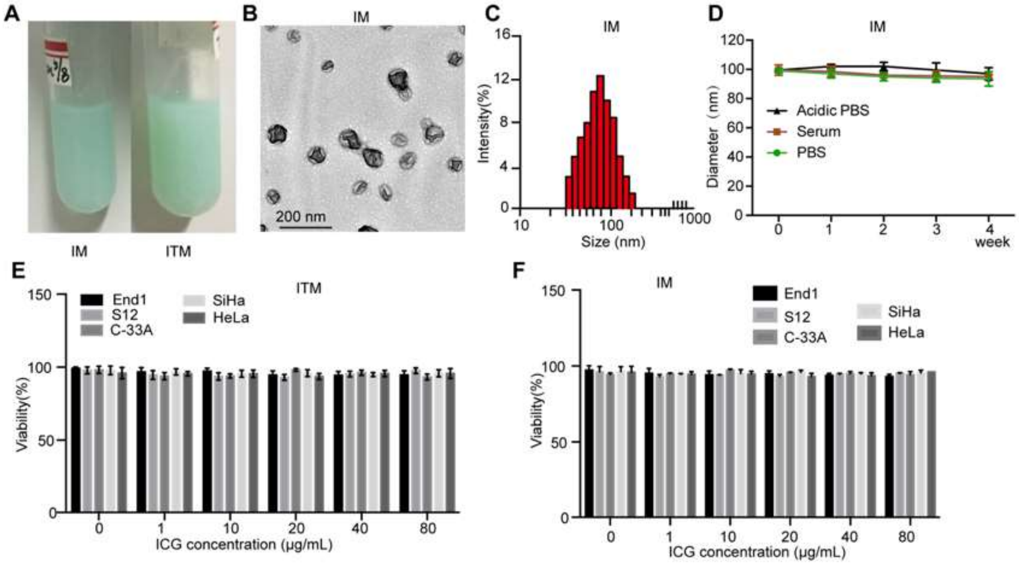

Figure S2: (A) The photograph of aqueous solution of ITM and IM. (B) TEM images of IM. The scale bars represent 200 nm. (C) The size distribution of IM by DLS. (D) Size stability test of IM stored in serum, in phosphate-buffered saline (PBS, pH=7.4) or in Acidic PBS (pH=5.4) in the dark at 25 °C. (E) Cytotoxicity of ITM toward cervical cells. (F) Cytotoxicity of IM toward cervical cells.

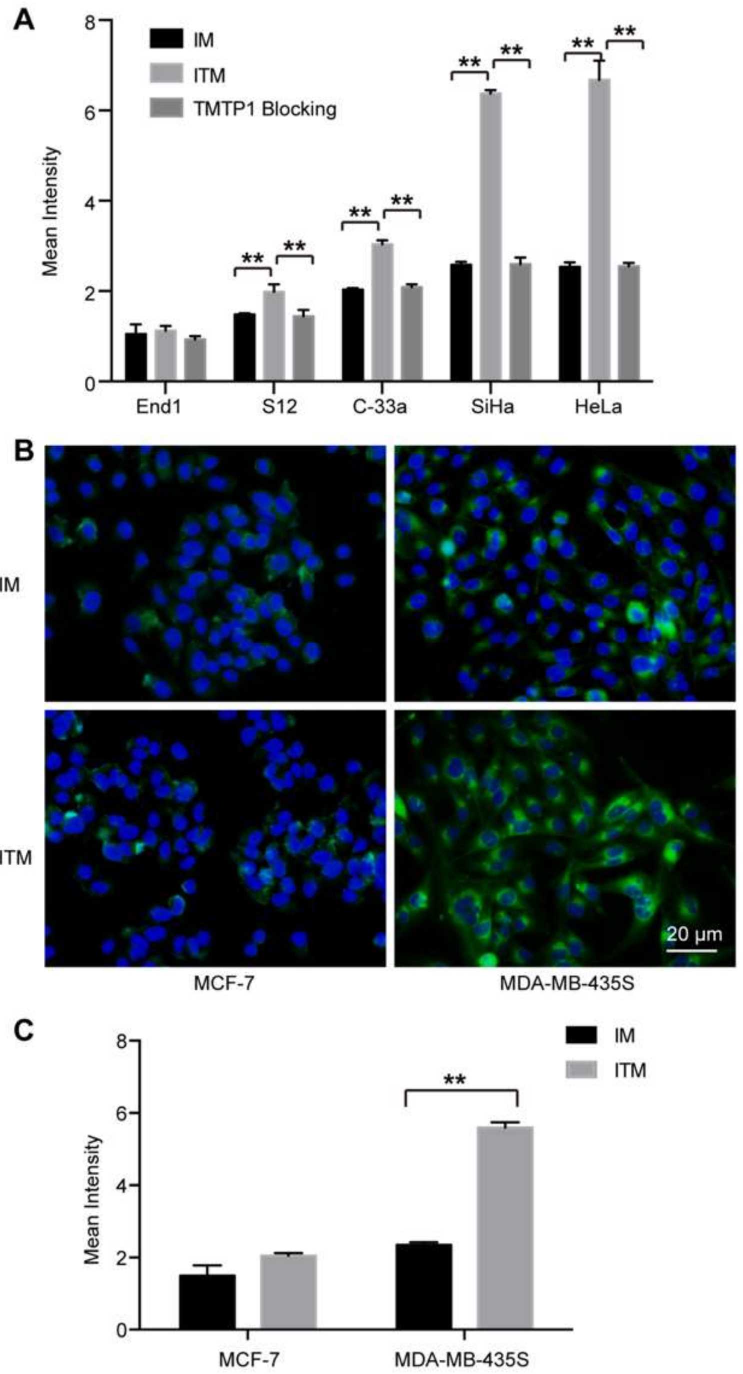

Figure S3: (A) Fluorescence intensity of cervical cells treated with IM and ITM (ICG concentration = 10 μg/mL) for 3 h was calculated by ImageJ software. (B) Confocal fluorescence images of MDA-MB-435S and MCF-7 treated with IM and ITM (ICG concentration=10 μg/mL) for 3 h *in vitro*. Blue represents the fluorescence of DAPI and green represents the molecules fluorescence of ICG. The fluorescence images were obtained at excitation wavelengths of 405 and 633 nm for DAPI and ICG,

respectively (scale bar, 20 μm). (C) Fluorescence intensity of MDA-MB-435S and MCF-7 was calculated by ImageJ software. (**P<0.01).

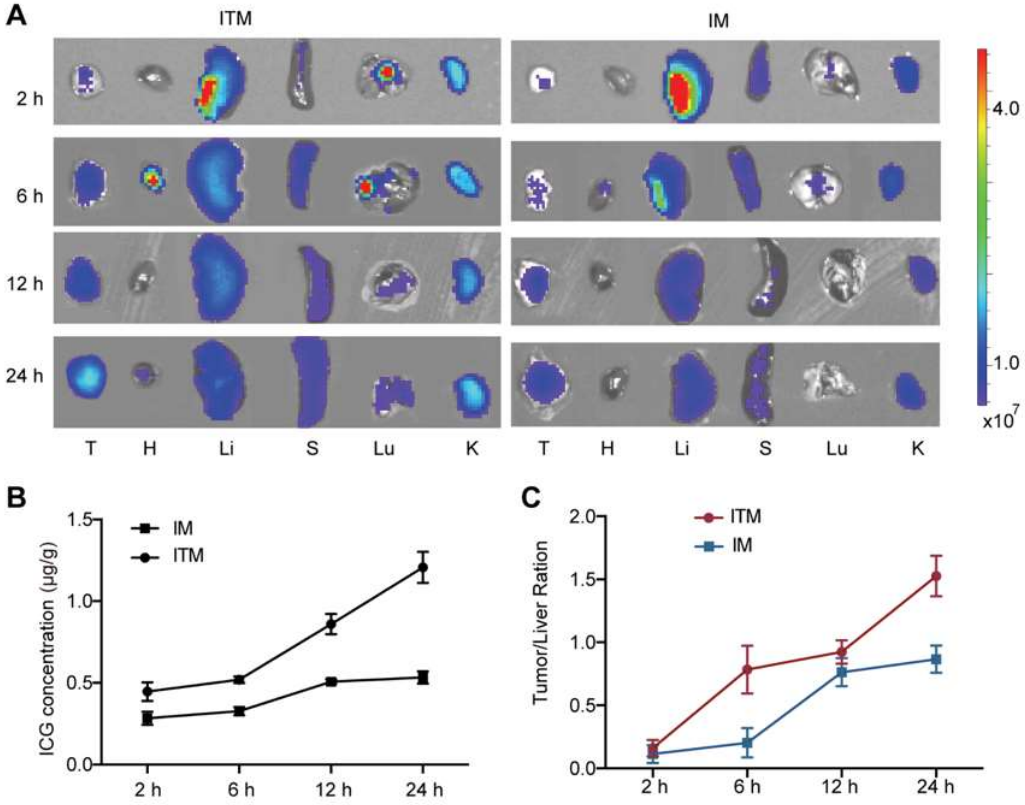

Figure S4: (A) NIR *ex-vitro* imaging of the main organs and tumor tissues after injection of ITM or IM at an ICG dose of 1.0 mg/kg at 2 h, 6 h, 12 h and 24 h (T, tumor; H, heart; Li, liver; S, spleen; Lu, lung; K, kidney). The NIR fluorescence signals were acquired at 840 nm with the excitation of 745 nm and were measured in radiance counts per cm^2 per second per steradian ($p/s/cm^2/sr$). (B) ICG concentration (Y-axis) in the tumor tissues (μg/g) of the athymic mice after injection of ITM or IM at an ICG dose of 1.0 mg/kg. (C) The tumor/liver ratio of ITM and IM after injection at 2 h, 6 h, 12 h and 24 h.

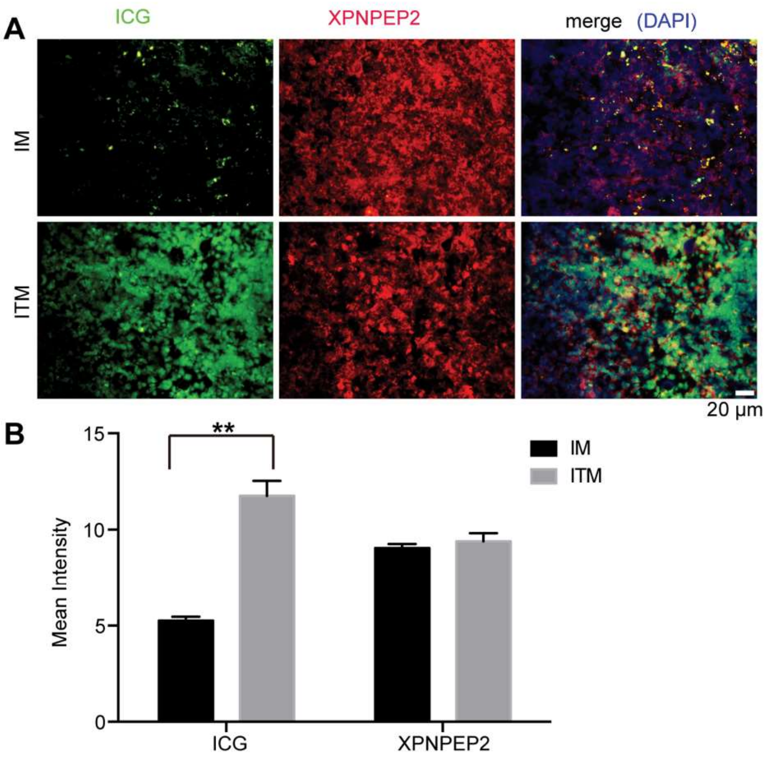

Figure S5. (A)Tumors of the mice with subcutaneous xenografts derived from HeLa cells after injection of ITM or IM (ICG dose, 1.0 mg/kg) at the time point of 48 h were sectioned into 6 μm slices and were then stained against XPNPEP2. Co-localization of XPNPEP2 (red) and ICG (green) was observed. Nuclei were stained as blue with DAPI. The fluorescence images were obtained at excitation wavelengths of 405 nm, 633 nm and 561 nm for DAPI, ICG and XPNPEP2, respectively (scale bar, 20 μm). (B) Fluorescence intensity of ICG and XPNPEP2 was calculated by ImageJ software. Data are expressed as the mean ± s.e.m. (**P<0.01).

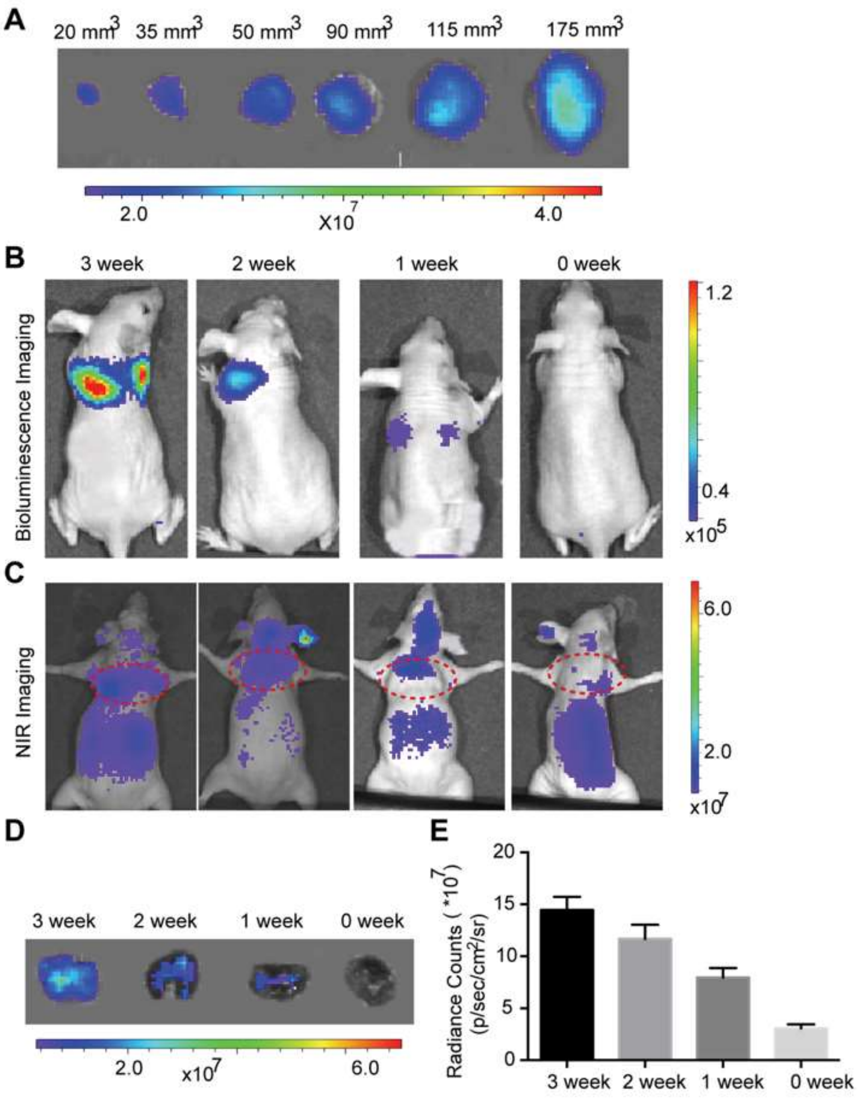

Figure S6. (A) *Ex-vitro* NIR imaging of the isolated tumors with different volumes after injection of ITM at an ICG dose of 1.0 mg/kg at 24 h. The NIR fluorescence signals were acquired at 840 nm with the excitation of 745 nm and were measured in radiance counts per cm^2 per second per steradian ($p/s/cm^2/sr$). (B) Bioluminescence imaging of lung metastasis foci in a lung metastasis model after inoculation of HeLa cells 0 week, 1 week, 2 week and 3 week. (C) *In vivo* and *ex vivo* (D) NIR imaging of lung metastasis foci at 24 h post ITM injection at an ICG dose of 1.0 mg/kg. (E) Average ICG fluorescence intensities of lung metastasis foci.

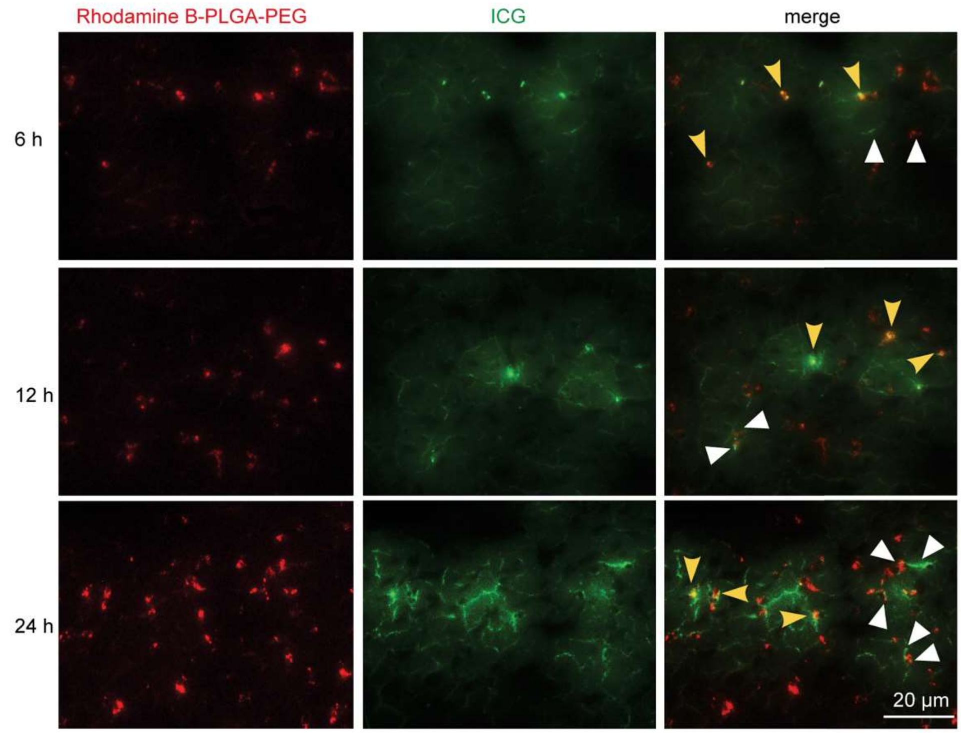

Figure S7. The different localization pattern of Rhodamine B labeled PLGA-PEG and ICG in the tumor over a 24 h period. The yellow arrows represent the overlap of red and green fluorescence. White arrows represent the separation of red and green fluorescence. The fluorescence images were obtained at excitation wavelengths of 633 nm and 555 nm for ICG and Rhodamine, respectively (scale bar, 20 μm).

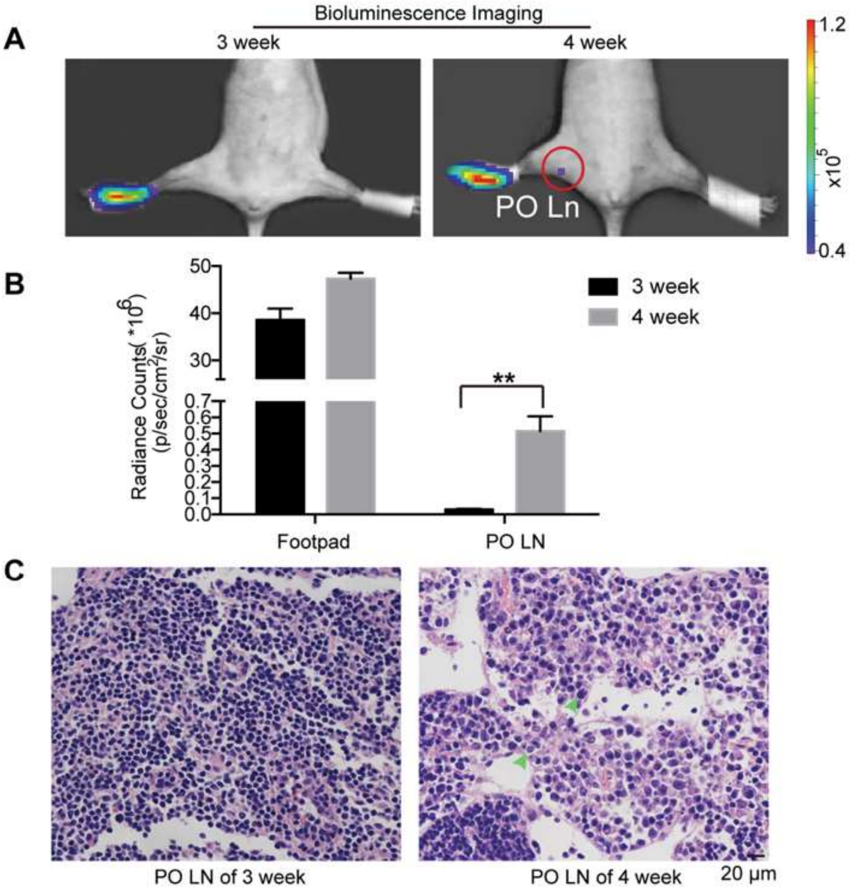

Figure S8**.** (A) Bioluminescence imaging of PO LN metastasis in HeLa-luc cell footpad planting mode at week 3 and at week 4. (B) The bioluminescence intensity of the footpad and PO LN at week 3 and week 4. (C) H&E staining of resected PO LN from the HeLa-luc cell foot pad planting mode at week 3 and at week 4. Green arrows represent the metastatic tumor cells (scale bar, 20 μm). Data are expressed as the mean ± s.e.m. (**P<0.01).

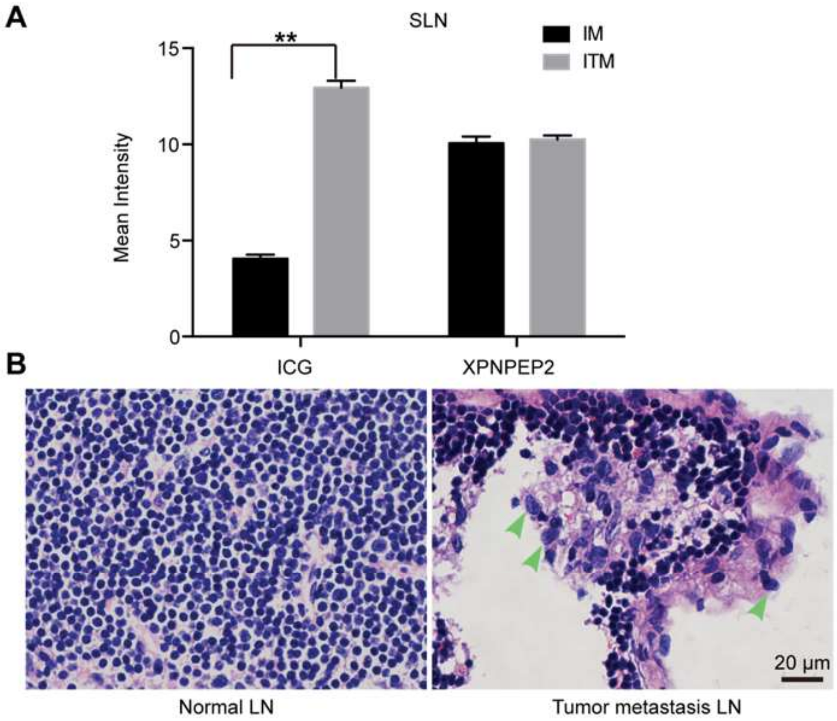

Figure S9: (A) Fluorescence intensity of ICG and XPNPEP2 in tumor metastasis SLN after injection of IM and ITM (ICG dose, 2 μg) was calculated by ImageJ software. (B) H&E staining of normal LN and the tumor metastasis LN. Green arrows represent the metastatic tumor cells (scale bar, 20 μm). Data are expressed as the mean ± s.e.m. (**P<0.01).

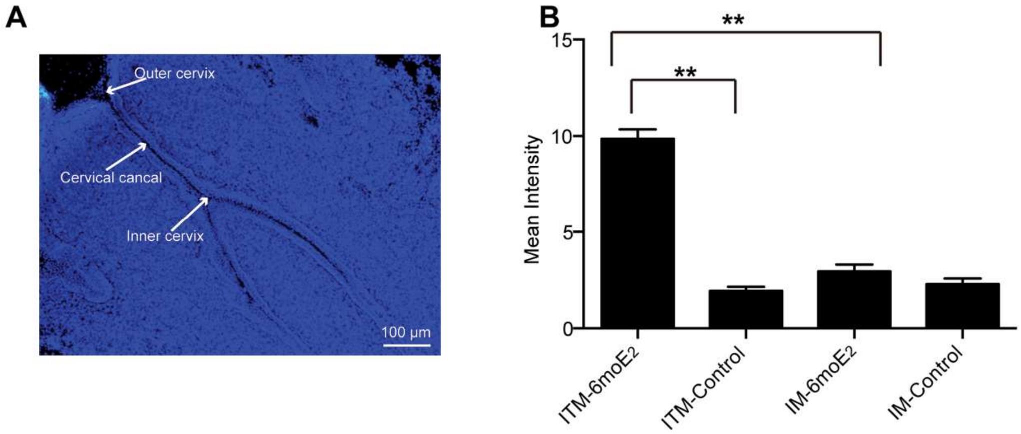

Figure S10. (A) Cervix were sectioned into 6 μm slices and stained against DAPI. (scale bar, 100 μm). (B) Quantitative analysis of ICG fluorescence intensity in the cervix epithelium 24 h post-injection with IM or ITM at a ICG dose of 1.0 mg /kg in 6mo E_2 group and control group was performed using ImageJ software. (**P<0.01).

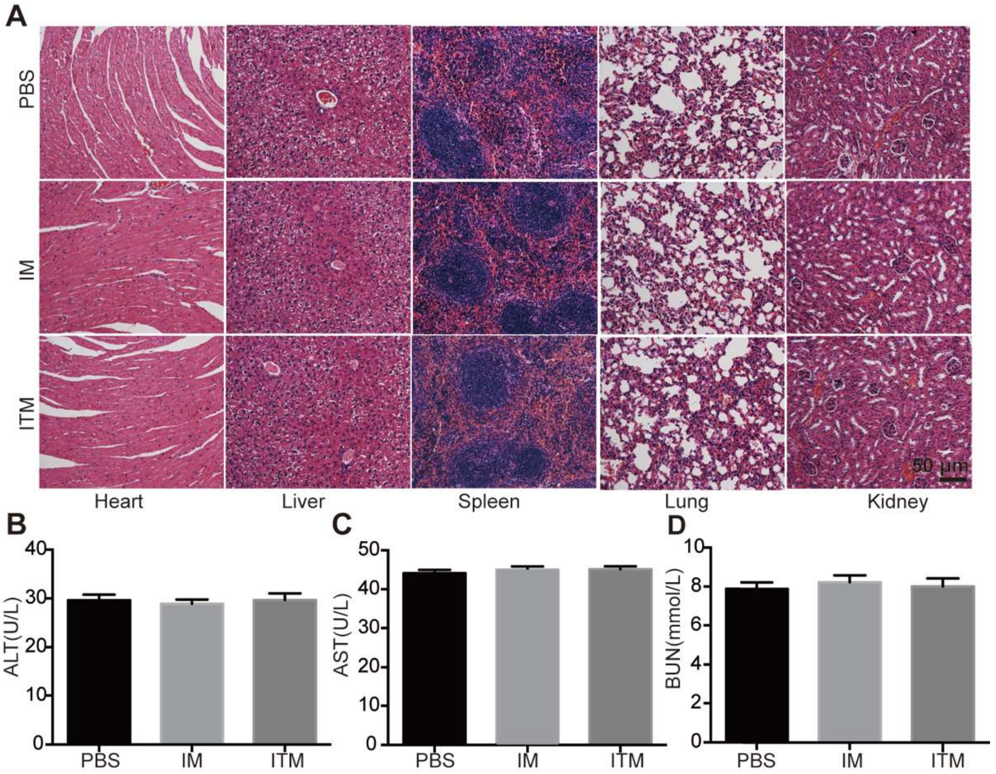

Figure S11. *In vivo* Toxicity of ICG-loaded micelles. (A) H&E staining of the heart, liver, spleen, lung and kidney from normal mice 28 days post injection of IM or ITM at a ICG dose of 10 mg /kg (scale bar, 50 μm). (B)(C) and (D) Quantitative analysis of ALT, AST and BUN in blood of each group of mice.

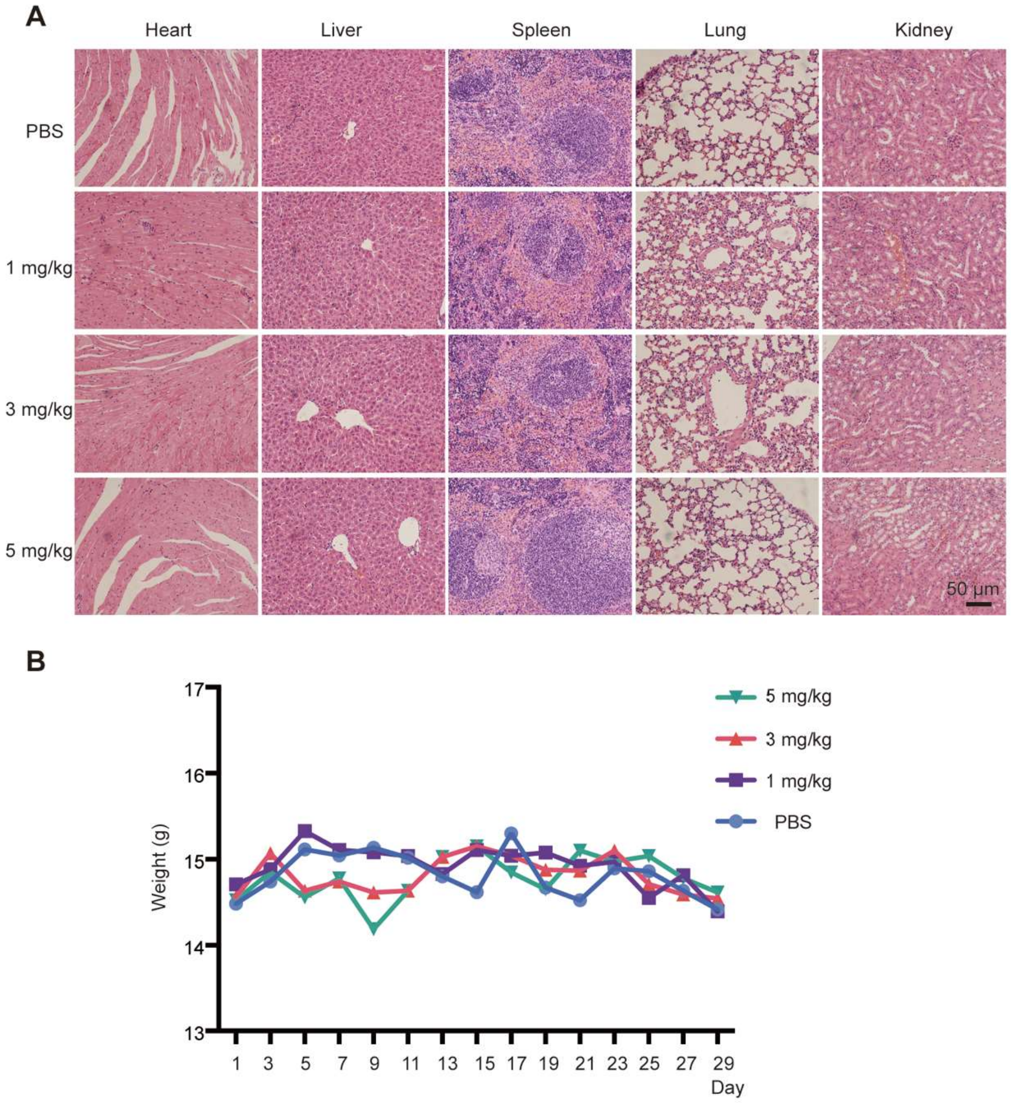

Figure S12**.** The repeat-dose toxicity study *in vivo*. (A) H&E staining of the heart, liver, spleen, lung and kidney from normal mice one month post injection of different doses (1 mg/kg, 3 mg/kg, 5 mg/kg) of ITM every day. The control group is received the same volume of sterile PBS. (scale bar, 50 μm). (B) Evolution of mice body weight following injection of different doses (1 mg/kg, 3 mg/kg, 5 mg/kg) of ITM or PBS within the month.

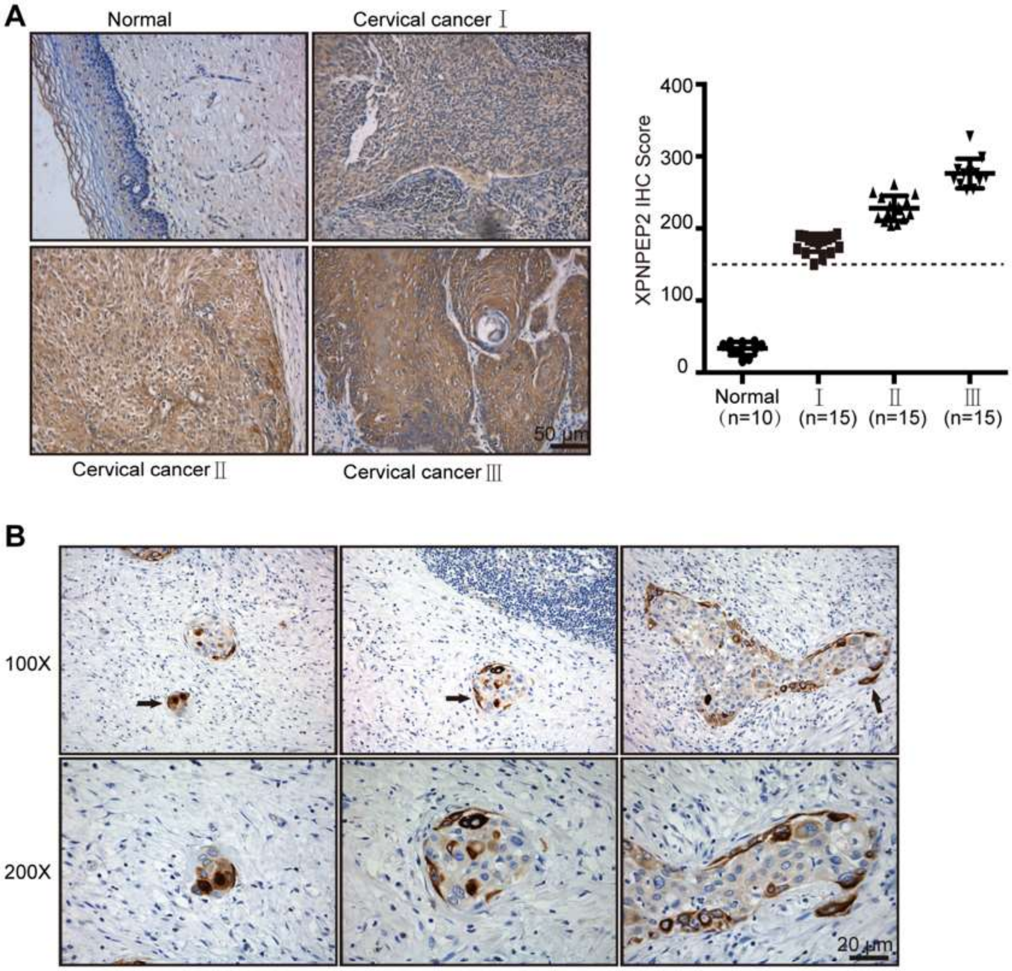

Figure S13**.** (A) IHC detection and scoring of XPNPEP2 in a series of cervical tissues, including normal cervical tissues (n=10), cervical cancer I (n=15), cervical cancer II (n=15) and cervical cancer III (n=15). (scale bar, 50 μm). (B) IHC detection of XPNPEP2 in micrometastases of cervical cancer. The black arrow refers to micrometastases. (scale bar, 20 μm).
